# Pharmacokinetic serum concentrations of VRC01 correlate with prevention of HIV-1 acquisition

**DOI:** 10.1016/j.ebiom.2023.104590

**Published:** 2023-06-08

**Authors:** Kelly E. Seaton, Yunda Huang, Shelly Karuna, Jack R. Heptinstall, Caroline Brackett, Kelvin Chiong, Lily Zhang, Nicole L. Yates, Mark Sampson, Erika Rudnicki, Michal Juraska, Allan C. deCamp, Paul T. Edlefsen, James I. Mullins, Carolyn Williamson, Raabya Rossenkhan, Elena E. Giorgi, Avi Kenny, Heather Angier, April Randhawa, Joshua A. Weiner, Michelle Rojas, Marcella Sarzotti-Kelsoe, Lu Zhang, Sheetal Sawant, Margaret E. Ackerman, Adrian B. McDermott, John R. Mascola, John Hural, M. Julianna McElrath, Philip Andrew, Jose A. Hidalgo, Jesse Clark, Fatima Laher, Catherine Orrell, Ian Frank, Pedro Gonzales, Srilatha Edupuganti, Nyaradzo Mgodi, Lawrence Corey, Lynn Morris, David Montefiori, Myron S. Cohen, Peter B. Gilbert, Georgia D. Tomaras

**Affiliations:** a Duke Center for Human Systems Immunology, Departments of Surgery, Immunology, Molecular Genetics and Microbiology, Durham, NC, 27710, USA; b Vaccine and Infectious Disease Division, Fred Hutchinson Cancer Center, Seattle, WA, 98109, USA; c Department of Global Health, University of Washington, Seattle, WA, 98195, USA; d Departments of Microbiology and Medicine, University of Washington, Seattle, WA, 98195, USA; e Division of Medical Virology, Institute of Infectious Disease & Molecular Medicine, University of Cape Town and National Health Laboratory Service, South Africa; f Department of Biostatistics, University of Washington, Seattle, WA, 98195, USA; g Thayer School of Engineering, Dartmouth College, Hanover, NH, 03755, USA; h Vaccine Research Center, Bethesda, MD, USA; i Family Health International, Durham, NC, 27710, USA; j Via Libre CRS, Lima, Peru; k Department of Medicine, Division of Infectious Disease and Department of Family Medicine in the David Geffen School of Medicine at UCLA, Los Angeles, CA, USA; l Perinatal HIV Research Unit (PHRU), Wits Health Consortium, Soweto, Johannesburg, South Africa; m Desmond Tutu Health Foundation, University of Cape Town (Institute of Infectious Disease and Molecular Medicine, and Department of Medicine), Observatory, 7925, Cape Town, South Africa; n Penn Center for AIDS Research, Infectious Disease Division, University of Pennsylvania, 3400 Civic Center Boulevard Building 421, Philadelphia, PA, 19104, USA; o Asociacion Civil Impacta Salud y Educación, San Miguel Clinical Research Center, Lima, Peru; p Division of Infectious Diseases, Emory University School of Medicine, Atlanta, GA, USA; q University of Zimbabwe-University of California San Francisco (UZ-UCSF) Collaborative Research Programme, Harare, Zimbabwe, South Africa; r Departments of Medicine and Laboratory Medicine, University of Washington, Seattle, WA, 98195, USA; s Division of Medical Virology, University of Cape Town, Anzio Road, Observatory, 7925, Cape Town, South Africa; t National Institute for Communicable Diseases, National Health Laboratory Service, Johannesburg, 2192, South Africa; u Antibody Immunity Research Unit, Faculty of Health Sciences, University of the Witwatersrand, Johannesburg, 2000, South Africa; v Centre for the AIDS Programme of Research in South Africa, University of KwaZulu-Natal, Durban, 4041, South Africa; w Institute of Global Health and Infectious Diseases, The University of North Carolina at Chapel Hill, Chapel Hill, NC, 27599, USA

**Keywords:** Body weight-based dosing, HIV, Prevention, Broadly neutralising antibodies

## Abstract

**Background:**

The phase 2b proof-of-concept Antibody Mediated Prevention (AMP) trials showed that VRC01, an anti-HIV-1 broadly neutralising antibody (bnAb), prevented acquisition of HIV-1 sensitive to VRC01. To inform future study design and dosing regimen selection of candidate bnAbs, we investigated the association of VRC01 serum concentration with HIV-1 acquisition using AMP trial data.

**Methods:**

The case–control sample included 107 VRC01 recipients who acquired HIV-1 and 82 VRC01 recipients who remained without HIV-1 during the study. We measured VRC01 serum concentrations with a qualified pharmacokinetic (PK) Binding Antibody Multiplex Assay. We employed nonlinear mixed effects PK modelling to estimate daily-grid VRC01 concentrations. Cox regression models were used to assess the association of VRC01 concentration at exposure and baseline body weight, with the hazard of HIV-1 acquisition and prevention efficacy as a function of VRC01 concentration. We also compared fixed dosing vs. body weight-based dosing via simulations.

**Findings:**

Estimated VRC01 concentrations in VRC01 recipients without HIV-1 were higher than those in VRC01 recipients who acquired HIV-1. Body weight was inversely associated with HIV-1 acquisition among both placebo and VRC01 recipients but did not modify the prevention efficacy of VRC01. VRC01 concentration was inversely correlated with HIV-1 acquisition, and positively correlated with prevention efficacy of VRC01. Simulation studies suggest that fixed dosing may be comparable to weight-based dosing in overall predicted prevention efficacy.

**Interpretation:**

These findings suggest that bnAb serum concentration may be a useful marker for dosing regimen selection, and operationally efficient fixed dosing regimens could be considered for future trials of HIV-1 bnAbs.

**Funding:**

Was provided by the 10.13039/100000002National Institutes of Health, 10.13039/100000060National Institute of Allergy and Infectious Diseases (NIAID) (UM1 AI068614, to the HIV Vaccine Trials Network [HVTN]; UM1 AI068635, to the HVTN Statistical Data and Management Center [SDMC], Fred Hutchinson Cancer Center [FHCC]; 2R37 054165 to the FHCC; UM1 AI068618, to HVTN Laboratory Center, FHCC; UM1 AI068619, to the HPTN Leadership and Operations Center; UM1 AI068613, to the HIV Prevention Trials Network [HPTN] Laboratory Center; UM1 AI068617, to the HPTN SDMC; and P30 AI027757, to the Center for AIDS Research, 10.13039/100006510Duke University (AI P30 AI064518) and 10.13039/100007812University of Washington (P30 AI027757) Centers for AIDS Research; R37AI054165 from NIAID to the FHCC; and OPP1032144 CA-VIMC 10.13039/100000865Bill & Melinda Gates Foundation.

**Research in context**

**Evidence before this study**

The Antibody Mediated Prevention (AMP) immunoprophylaxis trials, HVTN 704/HPTN 085 and HVTN 703/HPTN 081, tested the safety and HIV-1 prevention efficacy of the broadly neutralising antibody (bnAb) VRC01. These trials demonstrated efficacy against neutralization sensitive HIV-1 viruses when pooling across trials, although there was no overall HIV-1 prevention efficacy. Previous research using AMP trial data showed the predicted serum neutralization 80% inhibitory dilution titre (PT_80_) biomarker is a correlate of prevention efficacy, where PT_80_ is estimated as the ratio between serum concentration and the in vitro neutralization 80% (IC_80_) of VRC01 against the exposing virus. In addition, clinical trials have generally dosed HIV-1 bnAbs by body weight. Weight-based dosing has historically been assumed to account for participant differences in pharmacokinetics (PK). We searched PubMed using the search terms “HIV-1 antibody concentrations” AND “PK” AND “prevention” AND “efficacy.” Our search yielded no comparative trial.

**Added value of this study**

We found that baseline body weight was a potential confounder in the relationship between VRC01 concentration and HIV-1 acquisition, as body weight was associated with both VRC01 concentration and HIV-1 acquisition in AMP. However, VRC01 concentration remained inversely correlated with HIV-1 acquisition likelihood after accounting for VRC01 recipients’ body weight, suggesting that VRC01 concentration was an independent correlate of HIV-1 acquisition likelihood. We also found that VRC01 concentration was positively correlated with prevention efficacy of VRC01 against HIV-1 acquisition. Additionally, fixed dosing of a promising bnAb combination regimen had comparable overall predicted prevention efficacy as weight-based dosing in simulated AMP-like clinical trials.

**Implications of all the available evidence**

In the study design of future bnAb trials, bnAb serum concentration may be a useful marker for ranking dosing regimens with comparable neutralization profiles. In addition, future bnAb trials could consider fixed dosing, as opposed to body weight-based dosing, to reduce cost and operational complexity of administration.

## Introduction

Globally, 1.5 million people acquired HIV-1 in 2021.^1^ Although multiple HIV-1 prevention methods exist, uptake is limited by user preference and product availability contributing to a gap in the market for long-lasting, discreet, lifestyle-friendly methods.^2^ Therefore, broadly neutralising antibody (bnAb) immunoprophylaxis of HIV-1 is being researched.

During infection, the HIV-1 envelope gp120 binds with the human cell CD4 receptor and one of the human chemokine co-receptors CCR5 or CXCR4.^3^ On gp120, the CD4-binding site (CD4bs) is a highly conserved region. In pre-clinical research, monoclonal antibodies that target the CD4bs have demonstrated potent and effective neutralization against virus challenges in non-human primates.^4^, ^5^, ^6^, ^7^, ^8^ Several early phase clinical trials demonstrated safety, pharmacokinetics and functional responses of passively administered HIV-1 bnAbs.^9^^,^^10^ Only the VRC01 IgG1 antibody targeting the CD4bs has advanced to efficacy testing in two Antibody Mediated Prevention (AMP) trials. In AMP, the prevention efficacy of VRC01 was over 75% against VRC01-sensitive viruses, although there was no overall efficacy against all viruses.^11^ Previous research using AMP trial data showed that the predicted serum neutralization 80% inhibitory dilution titre (PT_80_) biomarker was a correlate of prevention efficacy, where PT_80_ is estimated as the ratio between serum concentration and the in vitro neutralization 80% (IC_80_) of VRC01 against the exposing virus.^12^

Here, we aimed to explore serum VRC01 concentration as a correlate of risk irrespective of the IC_80_ of VRC01 against the exposing virus. We hypothesized that the concentration correlate may be a useful marker when selecting bnAb dosing regimens for efficacy testing. In addition, since VRC01 dose amount was directly proportionate to body weight in AMP, we assessed the association between body weight and HIV-1 acquisition likelihood and explored body weight-based dosing vs. fixed dosing. We hypothesized that these two dosing regimens may achieve comparable prevention efficacy. Generally, HIV-1 bnAbs are dosed by body weight in clinical trials.^5^^,^^8^, ^9^, ^10^, ^11^, ^12^, ^13^, ^14^, ^15^, ^16^, ^17^, ^18^, ^19^, ^20^, ^21^, ^22^, ^23^, ^24^, ^25^, ^26^, ^27^, ^28^, ^29^ Weight-based dosing has historically been assumed to adjust for participant differences in pharmacokinetics (PK), yet body weight does not substantially affect monoclonal antibody (mAb) distribution and elimination as reported for oncology mAbs,^30^ and HIV-1 mAbs in adults.^17^^,^^18^ In AMP, VRC01 dose amount was directly proportionate to body weight, so we assessed the association between body weight and HIV-1 acquisition likelihood. These findings, combined with additional simulation studies, suggest that fixed dosing (vs. body weight-based dosing that current HIV-1 bnAb trials employ) may be effective and efficient.

## Methods

### Ethical compliance

All relevant ethical regulations were complied with. These analyses were approved by the Duke University Health System Institutional Review Board (Duke University) (protocol #Pro00093087). For the National Institute for Communicable Diseases the work was approved by the University of the Witwatersrand Human Research Ethics Committee (protocol #M201105). All participants in the AMP trials provided written informed consent.^11^

### Study procedures

#### Summary of AMP trials

HVTN 703/HPTN 081 (NCT02716675) and HVTN 704/HPTN 085 (NCT02568215), were harmonized Phase 2b randomized trials.^23^^,^^28^ Study protocols are available in the Supplemental Materials of Corey et al.^11^ AMP enrolled 4623 participants for ten intravenous (IV) infusions, randomized 1:1:1 to high dose VRC01 (30 mg/kg of body weight), low dose VRC01 (10 mg/kg of body weight), or placebo (saline) every eight weeks over 72 weeks. Study product dosing was calculated using the participant's body weight at the previous visit, unless body weight changed more than 10% on the day of the infusion visit. The primary endpoint, HIV-1 acquisition, was assessed over 80 weeks.

#### Case–control sampling

The two-phase case-control sampling design was previously described.^12^^,^^16^ Briefly, all VRC01 recipients diagnosed with HIV-1 by the week 80 visit were sampled for measurement of VRC01 serum concentrations at all blood storage visits (baseline, every four weeks through week 80-, and five-days post second infusion) through the last visit without HIV-1. These included 60 and 47 participants who acquired HIV-1 in the low (10 mg/kg) and high (30 mg/kg) dose arms, respectively; these participants received a median of six and four infusions of VRC01, respectively, prior to HIV-1 diagnosis.

Among VRC01 recipients completing the week 88 visit without acquiring HIV-1, a sample (restricted to those that did not permanently discontinue infusions) of 82 participants stratified by VRC01 dose arm, and geographic region (South America, USA/Switzerland, and sub-Saharan Africa) were selected to measure VRC01 concentration at all blood storage visits (baseline, 5 days post second infusion, every 4 weeks through Week 80, and Week 88). These included 40 participants from the low dose groups and 42 participants from the high dose groups with 89% receiving all ten infusions. Requiring these VRC01 recipients completing the week 88 visit without HIV-1 was to ensure that these participants did not acquire HIV-1 at Week 80, the same time frame for evaluating VRC01 recipients who acquired HIV-1 in the case–control study.

Since VRC01 recipients without HIV-1 had week 88 samples and VRC01 recipients who acquired HIV-1 were diagnosed with HIV-1 by week 80 (hence no week 88 samples), six VRC01 recipients without HIV-1 with their last trial visit occurring prior to week 88 were included to keep the lab blinded to the case–control status of each sample for unbiased assessments. Concentration data from these six participants were included in the PK modelling but were not included in the correlates analysis. Unless otherwise noted, all results are reported for the combined AMP trials.

### Laboratory methods

#### PK binding antibody multiplex assay (BAMA)

VRC01 bnAb concentrations were measured with a qualified anti-idiotype PK binding assay that was subsequently validated under the oversight of the Quality Assurance for Duke Vaccine Immunogenicity Programs using the same assay parameters. The PK-BAMA method demonstrated accuracy, precision, and specificity for detection of VRC01 in human serum samples.^29^ Accuracy of VRC01 IgG concentration measurements by PK-BAMA was evaluated by testing a blinded panel of 78 spiked samples with known VRC01 concentrations (0 mcg/ml–700 mcg/ml). Sample concentrations measured by the PK-BAMA method demonstrated excellent concordance with the true (known) concentration of VRC01 in serum.^12^ The VRC01 anti-idiotype PK assay was additionally qualified and validated for the following parameters: accuracy, precision, robustness and limits of detection and quantitation (Supplemental Figure S1). These results demonstrate that the PK-BAMA assay exhibits accurate quantification of VRC01 IgG bnAb for evaluation in the efficacy trial. Additional information about the PK BAMA assay is included in the Supplemental Methods.

VRC01 serum concentration estimates were calculated based on the VRC01 IgG monoclonal antibody standard curve (starting concentration 0.5 mcg/ml, titrated 3-fold for 11 total dilutions on the same plate) using the five-parameter logistic regression (5PL) equation. Additional assay controls and QC criteria are listed in the Supplemental Materials. The estimated concentrations were then multiplied by the dilution factor to estimate physiologic concentration. Limits of Detection and Quantitation were established in qualification experiments using commercially available HIV-1 seronegative serum samples (Supplemental Figure S1).

#### Neutralization assay

The optimized and subsequently validated^31^ TZM-bl target cell neutralization assay,^32^^,^^33^ was used to assess the IC_80_ in vitro sensitivity to VRC01 (in VRC01-recipient serum samples) of HIV-1 Env-pseudotyped viruses (for brevity, we refer to HIV-1 Env-pseudotyped viruses as “viruses” in the main text). Full details on pseudotyped virus stock preparation, the TZM-bl assay, and calculation of serum neutralization titres are reported in Gilbert et al.^12^

#### Anti-drug antibody assay

Anti-drug antibodies (ADAs) were detected and quantified using a qualified bridging electrochemiluminescence assay as previously described.^34^ Assays were conducted under Good Clinical Laboratory Practice (GCLP) standards. Samples were tested in duplicate along with a panel of anti-idiotype and negative controls and data were accepted based on meeting pre-established quality control criteria.

### Statistical methods

#### Population PK (popPK) modelling

Serum concentrations of VRC01 following intravenous (IV) administration were described by an open two-compartment disposition model with first-order elimination from the central compartment.^17^ The model was parameterized in terms of clearance from the central compartment (CL, L/day), volume of the central compartment (Vc, L), inter-compartmental distribution clearance (Q, L/day) and volume of the peripheral compartment (Vp, L). An exponential between-individual random effect was considered for CL, Vc, Q, and Vp based on patterns observed in the data. Nonlinear mixed effects modelling with the stochastic approximation of expectation-maximization (SAEM) estimation method was employed using Monolix software. Similar procedures described in Huang et al.^17^^,^^18^ were used to determine the final popPK model, which included total body weight (termed body weight) and trial (HVTN 704/HPTN 085 or HVTN 703/HPTN 081, termed study) as a covariate of CL. Of note, body mass index was also considered but removed from the final popPK model due to collinearity with body weight.

#### Baseline risk score

Within each AMP trial, the placebo group data were used to derive a baseline risk score (as a proxy of an individual's HIV-1 exposure) for every placebo and VRC01 recipient defined as the logit of predicted HIV-1 acquisition likelihood. Ensemble learning was used to build this risk score using as input variables: site, country, baseline age, and baseline values of the following: for HVTN 704/HPTN 085 – race, syphilis, smoking cigarettes or vaping, alcohol use, drug use (e.g., crack cocaine, amphetamine/methamphetamine, heroin, prescription pain killers), vaginal or anal sex within 2 h of drug use, IV infusion, use of condoms during vaginal or anal sex, vaginal or anal sex while drunk, and number of people they had vaginal or anal sex with; and for HVTN 703/HPTN 081 – gonorrhoea, chlamydia, smoking cigarettes, alcohol use, and drug use (e.g., amphetamine/methamphetamine, heroin, prescription pain killers, marijuana, ecstasy).

#### Concentration correlates

A Cox model using study enrolment (i.e., first study infusion date) as the time origin assessed the association of the current value of VRC01 serum concentration (included as a time-dependent covariate on a daily grid) with the instantaneous hazard of HIV-1 acquisition. To accommodate the case–control sampling design, we used empirical inverse probability sampling weights.^16^^,^^35^ HIV-1 acquisition dates were estimated based on an algorithm that integrated the HIV-1 diagnostic information and the gag-pol viral sequence information.^12^^,^^36^^,^^37^ The popPK model was used to estimate each participant's VRC01 time-concentration curve over a daily grid, and for each VRC01 recipient who acquired HIV-1 the estimated concentration at exposure was calculated by evaluating this curve at the estimated HIV-1 acquisition date. Regression calibration was used to account for measurement error in these estimates. The Cox model was adjusted for trial (HVTN 704/HPTN 085 or HVTN 703/HPTN 081) and dose group (10 mg/kg or 30 mg/kg). Adjusting for additional baseline covariates including body weight and risk score was also considered in supportive analyses. The concentration correlate analysis was also repeated by the IC_80_ value (≤3.0 or >3.0 mcg/ml) of VRC01 against the exposing virus of the VRC01 recipients who acquired HIV-1. In the analyses restricting to IC_80_ ≤ 3.0 mcg/ml, the event time was right censored at the estimated time of HIV-1 acquisition for VRC01 recipients who acquired HIV-1 with IC_80_ > 3.0 mcg/ml; in the analyses restricting to IC_80_ > 3.0 mcg/ml, the event time was right censored at the estimated time of HIV-1 acquisition for VRC01 recipients who acquired HIV-1 with IC_80_ ≤ 3.0 mcg/ml. All VRC01 recipients without HIV-1 were included in both analyses. We used a nonparametric bootstrap procedure to compute 95% confidence intervals (CIs) for the hazard ratio (HR) of acquired HIV-1 per ten-fold increment in VRC01 concentration. These CIs were inverted to compute a two-sided P-value for whether VRC01 concentration correlated with HIV-1 acquisition. The relationship between predicted VRC01 serum concentration at the time of exposure and prevention efficacy was estimated with a log-linear curve using Cox regression model of data from both VRC01 and placebo recipients. The hazard function is expressed as $h\left(t\right)=h_{0}\left(t\right)\;\exp\left\{Z\left[\beta_{0}+\beta_{1}*C\left(t\right)\right]\right\}$ and prevention efficacy as $PE\left(C\left(t\right)\right)=1-\exp\left\{\beta_{0}+\beta_{1}*C\left(t\right)\right\}$, where $h_{0}\left(t\right)$ is the likelihood of HIV-1 at time *t* for a placebo recipient, Z is 1 for those in the VRC01 arms and 0 otherwise, *C(t)* is log_10_ VRC01 serum concentration at time *t*, $1-\exp\left\{\beta_{0}\right\}$ is PE (C(t) = 0), and $\exp\left\{\beta_{1}\right\}$ is the hazard ratio of HIV-1 per log_10_ increase in VRC01 serum concentration, under the assumption that PE(C(t) ≤ c) = 0 where c is chosen to be a small number indicating a non-effective concentration level of VRC01. The corresponding relationship between PT_80_ at exposure and prevention efficacy was derived by calculating PT_80_ as the ratio between serum concentration at exposure and the geometric mean IC_80_ of VRC01 against all exposing viruses. A nonparametric bootstrap procedure was used to compute pointwise 95% CIs (bounded above zero) for the estimated prevention efficacy at each given VRC01 concentration and PT_80_ value.

#### Sensitivity analysis

To assess the impact of HIV-1 acquisition date estimates on the correlates finding, we conducted a sensitivity analysis by varying the estimated date of HIV-1 acquisition for all VRC01 recipients who acquired HIV-1 when examining the association of VRC01 serum concentration with the instantaneous hazard of HIV-1 acquisition. Specifically, we repeated the Cox model analysis described above 20 times using, for each VRC01 recipient who acquired HIV-1, randomly sampled HIV-1 acquisition dates uniformly distributed between their HIV-1 diagnosis date and two trial visits prior with the latter being a conservative lower bound for their last visit prior to HIV-1 acquisition.

#### Body weight association with HIV-1 acquisition

Since VRC01 dosing is body weight dependent and serum concentration levels were different between VRC01 recipients who acquired HIV-1 and those without HIV-1, we assessed the association between baseline body weight and HIV-1 acquisition likelihood in a Cox regression (semi-parametric) model, with and without adjustment for baseline risk score. We performed the analyses separately in the placebo and the VRC01 groups within each AMP trial and pooled, based on data from the entire trial population without HIV-1 at enrolment from both AMP trials (n = 4611).^11^ We also used a nonparametric estimator, controlling for potential confounders (age, risk score, and trial) to assess the association. The latter is an adaptation of the causal isotonic regression method,^38^ which assumes the true curve is monotonically decreasing to a setting in which the outcome is right-censored without relying on any parametric form of the relationship. The output of the method is a step function, even if the true underlying function is smooth. We truncated the curve estimates at the 5% and 95% percentiles of the body weight distribution since the estimates can be unstable towards the tails.

#### Predicted prevention efficacy of fixed vs. body weight-based dosing

As body weight was predictive of likelihood of HIV-1 acquisition and an individual's bnAb concentration is proportionate to body weight at a given time post administration, we compared fixed dosing vs. body weight-based dosing via a simulation of 1000 AMP-like trials. In addition to looking at the fixed vs. body weight-based dosing for VRC01, we conducted simulation studies using the triple bnAb combination (VRC07-523LS + PGT121LS + PGDM1400LS) as future trials will not use VRC01 alone and this combination is one of the most promising regimens in the pipeline. The prediction of prevention efficacy for the triple bnAb combination was carried out using the same approach described in Gilbert et al.^12^ In the simulation of serum concentrations of each bnAb over time, the fixed and body weight-based dosing regimens were compared while fixing the total dose amount aggregated across all individuals to be the same between the two regimens. For the fixed dosing regimen, every individual in the simulated trials received 2 g or 2.3 g of VRC01 and 2.7 g + 2.7 g + 2.7 g or 3.1 g + 3.1 g + 3.1 g of the triple bnAbs, where the lower fixed dose amount was for settings with AMP HVTN 703/HPTN 081-like trial participants who had a lower average body weight, and the higher fixed dose amount was for settings with HVTN 704/HPTN 085-like trial participants who had a higher average body weight. For the body weight-based dosing regimen, every bnAb recipient received a dose amount proportionate to their body weight at 30 mg/kg for VRC01 and at 40 mg/kg + 40 mg/kg + 40 mg/kg for the triple bnAbs. The body weight of individuals in the simulated trials were sampled from those in the two AMP trials pertaining to each trial population.

### Role of the funding source

The content of this manuscript is solely the responsibility of the authors and does not necessarily represent the official views of the National Institutes of Health. The funders had no role in study design, data collection and analysis, decision to publish, or preparation of the manuscript. Drs. Seaton, Huang and Tomaras (corresponding authors) had final responsibility for the decision to submit for publication; Dr. Yunda Huang had full access to all the data in the study.

## Results

### Serum concentration and popPK model

**Fig. 1:**
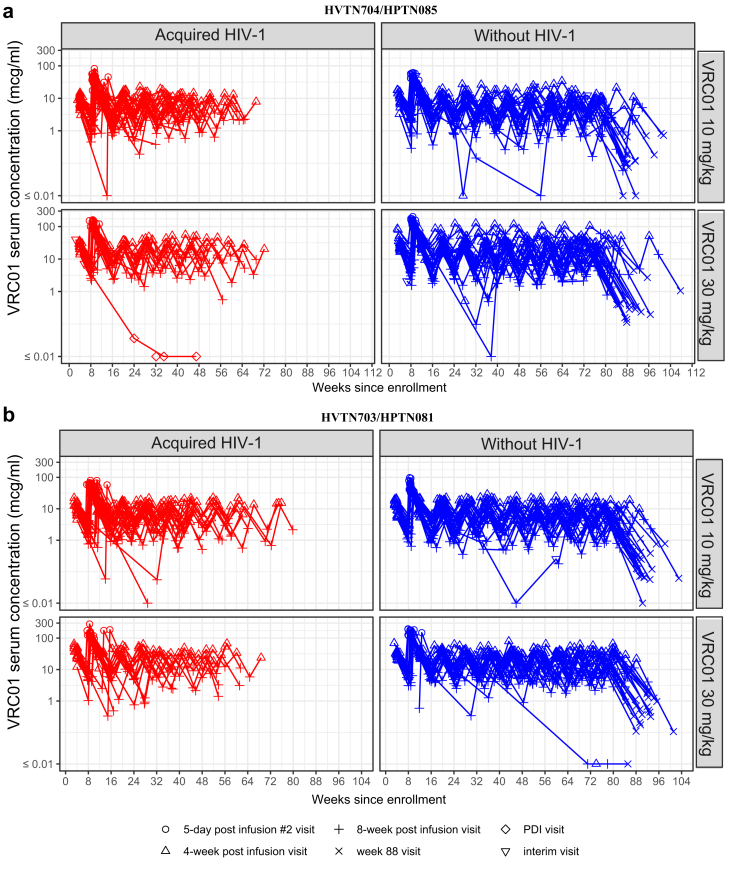
**Serum VRC01 concentration (log_10_) kinetics between cohorts and across infusion intervals.** Antibody concentrations per participant are shown over time since enrolment by dose group (10 mg/kg and 30 mg/kg) and case–control status (acquired HIV-1/without HIV-1) in (**a**) HVTN 704/HPTN 085 (n = 104) and (**b**) HVTN 703/HPTN 081 (n = 91). Red symbols and lines indicate VRC01 recipients that acquired HIV-1 and blue lines and symbols indicate VRC01 recipients without HIV-1.

Observed VRC01 serum concentrations were highest five days after the second infusion (compared to other 4-weekly measurements), with a median of 48.4 and 143.5 mcg/ml in the low and high dose groups, respectively. Observed VRC01 serum concentrations were lowest 16 weeks after the last infusion, reaching levels below the lower limit of quantitation of the assay (0.07 mcg/ml), especially in the low dose group (Fig. 1). The median observed VRC01 serum concentrations over time and across VRC01 recipients without HIV-1 in the case–control cohort was 10.1 mcg/ml and 31.0 mcg/ml in the 10 mg/kg and 30 mg/kg dose groups, respectively (Table 1). VRC01 serum concentration patterns were steady over successive infusions with no evidence of inter-infusion variations in key pharmacokinetics parameters (Supplemental Figure S2a–d). Among a randomly drawn set of 200 participants without HIV-1, 3% exhibited ADA responses with titres ranging from 1 to 81 at one or more post-infusion timepoints, and 97% were ADA negative (Supplemental Figure S3).

**Table 1:** Median observed VRC01 bnAb concentrations among recipients without HIV-1 by dosing regimen and by trial.

AMP trial	Median VRC01 serum concentration		
	10 mg/kg	30 mg/kg	VRC01 pooled
HVTN 704/HPTN 085	9.3 mg/ml (n = 19)	28.4 mg/ml (n = 21)	18.7 mg/ml
HVTN 703/HPTN 081	12.2 mg/ml (n = 21)	32.1 mg/ml (n = 21)	20.5 mg/ml
Pooled	10.1 mg/ml	31.0 mg/ml	19.6 mg/ml

The values are calculated from the subset of case-control data set for VRC01 recipients without HIV-1, as medians of participant-specific medians over all mid-infusion visits through to the week 76 visit (n=82).

The number of recipients without HIV-1 included in subsequent analyses is listed in parentheses for each group.

The final popPK model fitted the data well and offered satisfactory goodness-of-fit diagnostics (Supplemental Figure S4, Supplemental Figure S5a–c). Specifically, study was a significant predictor of both CL and Vp with higher values in HVTN 704/HPTN 085 than in HVTN 703/HPTN 081, leading to comparable elimination half-lives between the two studies. In addition, body weight was a significant positive predictor of CL (Supplemental Figure S4). The daily grid serum concentrations for participants in the case–control cohort were estimated based on this final popPK model.

### Concentration correlates

**Fig. 2:**
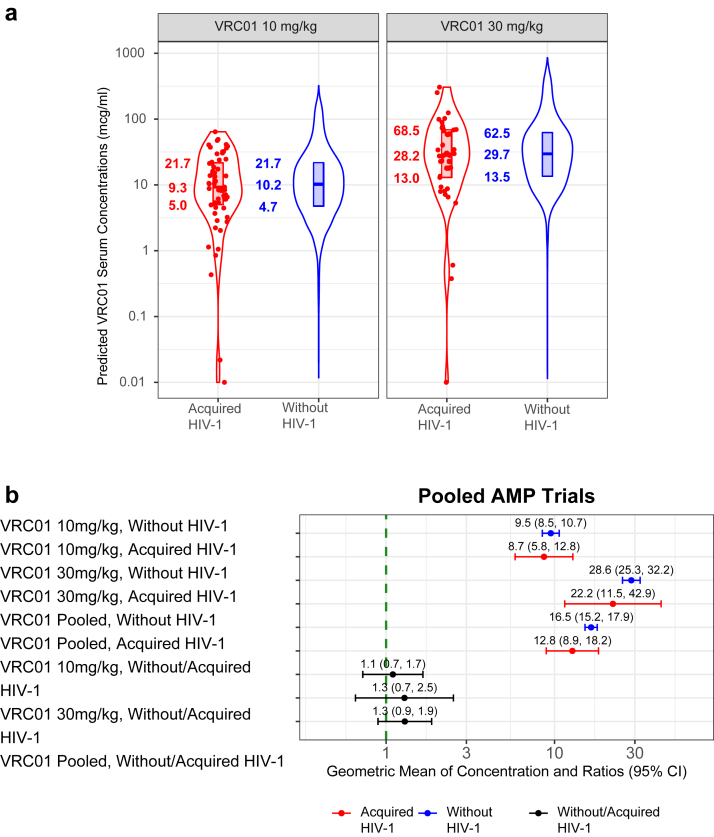
**VRC01 serum concentration association with instantaneous likelihood of HIV-1 acquisition.** (**a**) Violin plots of predicted concentration at estimated time of HIV-1 acquisition in VRC01 recipients who acquired HIV-1 and estimated daily concentration day 1 through week 80 in VRC01 recipients without HIV-1 (n = 189) (**b**) Geometric mean of serum concentration, geometric mean ratios, and 95% CI are shown in red for VRC01 recipients who acquired HIV-1 and in blue for VRC01 recipients without HIV-1 for the high and low dose groups (combined across trials) (n = 189). The concentration ratio (controls/cases) and 95% CI for each dose group (10 mg/kg and 30 mg/kg) and pooled across dose groups are shown in black. For each VRC01 recipient who acquired HIV-1, these calculations use the average (log-transformed) daily concentration weighted by the Bayesian posterior distribution of daily probabilities of HIV-1 acquisition over the entire grid of possible dates of HIV-1 acquisition. For each VRC01 recipient without HIV-1, these calculations use their median estimated daily concentration from Day 1 to week 80.

A comparison of PK parameters, including distribution and elimination half-life estimates from the final popPK model indicated no significant differences between VRC01 recipients who acquired HIV-1 and those without HIV-1 (Supplemental Figure S6a and b). However, estimated daily grid VRC01 serum concentrations in VRC01 recipients without HIV-1, as representative values at random HIV-1 exposure times, were higher than the estimated concentrations at the estimated time of acquisition for VRC01 recipients who later acquired HIV-1 in both dose groups, with a median of 10.2 vs. 9.3 mcg/ml in the 10 mg/kg dose group, and 29.7 vs. 28.2 mcg/ml in the 30 mg/kg dose group (Fig. 2a). In the dose-pooled group, although the trend was not statistically significant, the geometric mean of the individual-specific time-averaged median concentration of VRC01 recipients without HIV-1 was higher compared to the geometric mean of the estimated concentration at exposure in VRC01 recipients who acquired HIV-1 (geometric mean ratio [GMR] 1.3, 95% CI 0.9–1.9). The same trend was observed in both dose groups (10 mg/kg: GMR 1.1, 95% CI 0.7–1.7; 30 mg/kg: GMR 1.3, 95% CI 0.7–2.5) (Fig. 2b). Although the CI crosses 1.0 in this analysis, the outcome is consistent with our finding of statistically significant reduction in the likelihood of HIV-1 acquisition per log_10_ increase in VRC01 concentrations by the Cox model analyses.

**Fig. 3:**
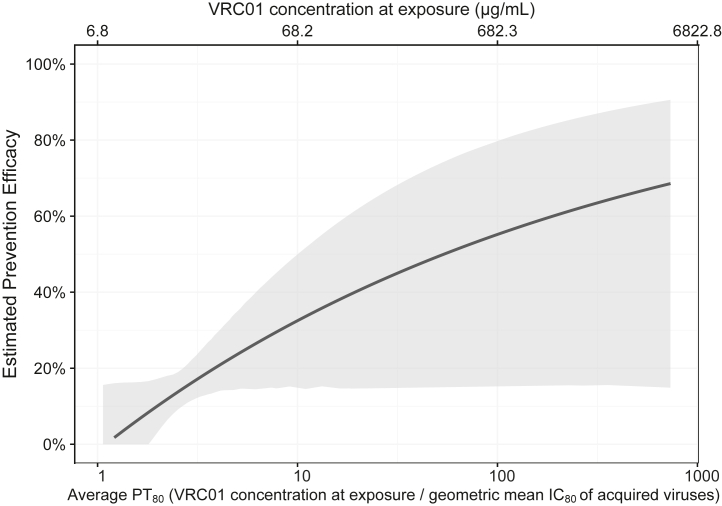
**Prevention efficacy (PE) of VRC01 concentrations against HIV-1 acquisition as a function of VRC01 concentration at exposure (top x-axis) and of average predicted PT**_**80**_**neutralization titre at exposure (bottom x-axis).** Shaded area provides 95% pointwise confidence intervals. Prevention efficacy at a given level of VRC01 concentration or PT_80_ was estimated via Cox regression models by pooled treatment group and dose. The geometric mean IC_80_ used to convert VRC01 concentration to PT_80_ was 6.8 μg/ml.

The overall inverse association between the current value of VRC01 serum concentration at HIV-1 exposure and likelihood of HIV-1 acquisition was also found in the virus-phenotype-specific analyses: the hazard ratio (HR) per-log_10_ increase of concentration was 0.81 (95% CI 0.47, 1.40) and 0.51 (95% CI 0.25, 1.02), respectively, when restricting to acquisition events with IC_80_ > 3.0 mcg/ml viruses or with IC_80_ ≤ 3.0 mcg/ml viruses. The positive association between VRC01 serum concentration at exposure as well as the corresponding PT_80_ against a representative virus and prevention efficacy is shown in Fig. 3.

**Fig. 4:**
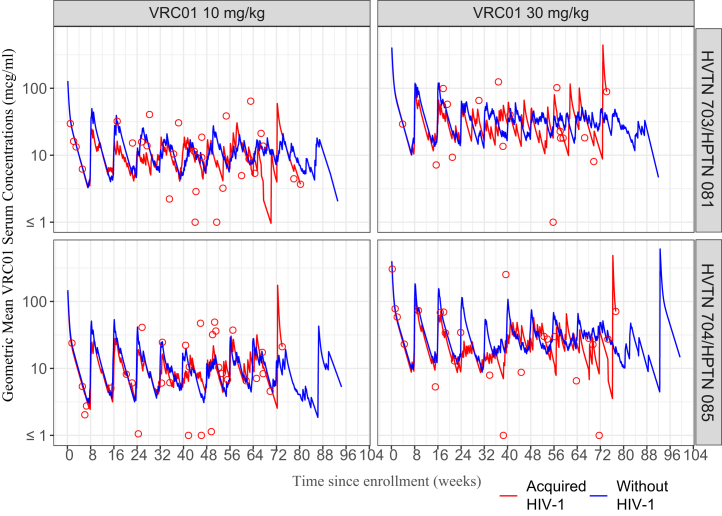
**VRC01 concentrations over-time in VRC01 recipients who acquired HIV-1 and VRC01 recipients without HIV-1.** Geometric mean VRC01 serum concentrations in the low dose (10 mg/kg) and high dose (30 mg/kg) in HVTN 703/HPTN 081 (top panels) and HVTN 704/HPTN 085 (lower panels) through week 80 post enrolment. VRC01 recipients who acquired HIV-1 (n = 107) are indicated in red curves, with open red circles. VRC01 recipients without HIV-1 (n = 82) are indicated in blue curves. For each VRC01 recipient without HIV-1, their geometric mean concentration calculated across the grid of daily concentration values through week 80 was used in the calculation. For each VRC01 recipient who acquired HIV-1, their estimated concentration at the estimated date of HIV-1 acquisition was used in the calculation.

In addition, the geometric mean concentration of VRC01 recipients who acquired HIV-1 at all times prior to any evidence of infection (not only at the estimated time of acquisition) appeared to be lower than the average concentration of VRC01 recipients without HIV-1 (Fig. 4). In many VRC01 recipients who acquired HIV-1, estimated serum concentrations were high at the estimated time of acquisition. This may be due to bnAb resistance to the infecting strain, or that the acquisition timing confidence interval spans the date of infusion. This pattern was consistent with the sensitivity analysis result that using “uniform-draw” acquisition time estimates, the median inter-quartile range hazard ratio estimate across the 20 datasets was 0.53 (0.30–0.88) (data not shown).

### Body weight

**Fig. 5:**
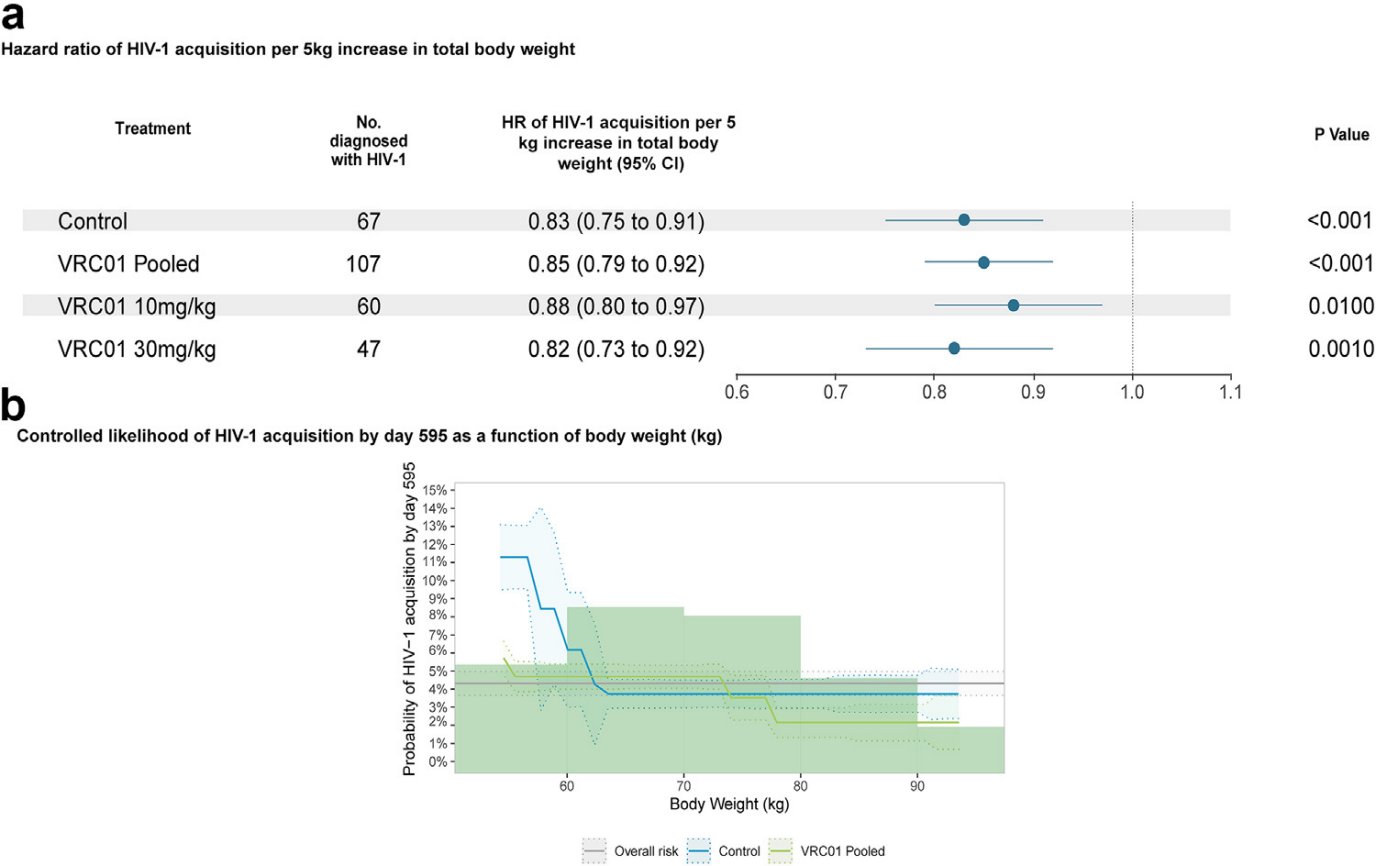
**HIV-1 acquisition and body weight.** (**a**) Hazard ratio of HIV-1 acquisition per 5 kg increase in total body weight. Hazard ratio of HIV-1 acquisition per 5 kg increase in participants' baseline body weight was estimated via Cox regression models by pooled treatment group and dose (n = 4611) (**b**) Controlled risk of HIV-1 acquisition by day 595 as a function of body weight (kg) (n = 4611).

Since body weight was a significant predictor of a key PK parameter and hence of individuals' VRC01 serum concentrations over time, we also investigated its association with the study outcomes. We found that participants’ body weight was significantly predictive of HIV-1 acquisition likelihood among both placebo recipients (HR = 0.83 per 5 kg increase in body weight, 95% CI 0.75, 0.91, P < 0.001) and VRC01 recipients (HR = 0.85, 95% CI 0.79, 0.92, P < 0.001) in the two AMP trials pooled; these were assessed by the Cox regression models that assume a continuous association between body weight and log-transformed hazard of HIV-1 (Fig. 5a). Supplemental Figure S7 shows the hazard ratios of HIV-1 acquisition per 5 kg increase in body weight for each trial, separately. Additional non-parametric analyses that relaxed the above assumption also showed such an association, where there was a decrease in HIV-1 acquisition likelihood with greater body weight (Fig. 5b, Supplemental Figure S8). These results suggest that body weight was a potential confounder of the correlate analysis; we found that the VRC01 serum concentration correlate remained significant after adjusting for body weight, with an estimated hazard ratio of HIV-1 acquisition per 10-fold increase in VRC01 concentration of 0.56 (95% CI 0.32–0.99, P = 0.042).

### Predicted prevention efficacy of fixed vs. body weight-based dosing

**Fig. 6:**
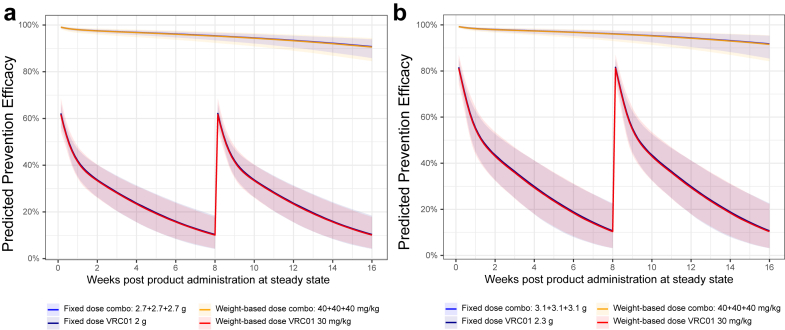
**Predicted prevention efficacy of fixed dose versus body weight-dose of the triple bnAb regimen PGT121LS + PGDM1400LS + VRC07-523LS and of VRC01 at steady state against** (**a**) HVTN703/HPTN 081 placebo viruses (n = 47) and (**b**) HVTN704/HPTN 085 placebo viruses (n = 70) circulating in each AMP trial. All predictions were made under the scenario that PGT121LS and PGDM1400LS have 5-fold higher half-lives than PGT121 and PGDM1400, based on modelling of the observed serum concentration data of PGT121^27^ and PGDM1400.^19^ Predicted prevention efficacy at steady state was based on prevention efficacy vs. PT_80_ curve in AMP^12^ and Pegu et al.,^5^ solid line: median. Shaded area: 95% prediction interval.

We compared the predicted prevention efficacy of VRC01 and the triple bnAb combination VRC07-523LS + PGT121LS + PGDM1400LS, when given either at a fixed dose or a body weight-based dose for each individual in simulated AMP-like trials. With the total dose amount of the bnAb regimens across all individuals being kept the same in each simulated trial, we found that fixed dosing and body weight-based dosing regimens had a comparable overall predicted prevention efficacy on a population level (Fig. 6, Supplemental Figure S9). Of note, individuals with body weight above or below the average may have altered HIV-1 acquisition likelihood between the fixed versus body weight-based dosing.

## Discussion

Based on analyses of AMP trial data, we present three main findings which can inform future bnAb engineering and dosing. First, estimated VRC01 concentrations in VRC01 recipients without HIV-1 were higher than those in VRC01 recipients who acquired HIV-1, and serum VRC01 concentration was positively correlated with the prevention efficacy of VRC01. Second, participants' body weight was inversely associated with HIV-1 acquisition among placebo and VRC01 recipients, separately, but serum VRC01 concentration remained inversely correlated with HIV-1 acquisition likelihood after accounting for VRC01 recipients’ body weight. Third, fixed-dosing had comparable overall predicted prevention efficacy as body weight-based dosing of a bnAb combination regimen in simulation studies.

Similar to Gilbert et al.,^12^ we found a statistically significant reduction in the likelihood of HIV-1 acquisition per unit increase in the current value of VRC01 serum concentrations at exposure among VRC01 recipients in AMP. In addition, sensitivity analyses showed the same trend and the concentration correlate remained statistically significant when the estimated time of infection was randomly varied for VRC01 recipients who acquired HIV-1. There was a consistent trend across both dose groups and pooled, although the difference in average concentrations between VRC01 recipients who acquired HIV-1 and those without HIV-1 was not statistically significant. This was likely due to the limited number of VRC01 recipients who acquired HIV-1 and the relatively large variation in the estimated concentrations at the estimated time of acquisition among VRC01 recipients who acquired HIV-1; such variation was primarily due to uncertainties in the estimated time of infection based on the infrequent 4-weekly HIV-1 diagnosis visits in the study. Importantly, the inverse association between serum VRC01 concentration and HIV-1 acquisition held when analysis was restricted to exposing viruses that were either sensitive (IC_80_ ≤ 3.0 mcg/ml) or resistant (IC_80_ > 3.0 mcg/ml) to VRC01 via neutralization. Specifically, higher serum VRC01 concentrations correlated with lower HIV-1 acquisition likelihood regardless of the neutralization sensitivity of the exposing virus to VRC01. This association appeared to be less apparent in the analysis restricting to resistant exposing viruses, likely because concentration plays a smaller role when the exposing viruses are resistant. This observation is consistent with the reported association between HIV-1 acquisition and the predicted PT_80_ neutralization titre as a ratio between concentration and IC_80_.^12^

In addition, we also reported that higher serum VRC01 concentration at exposure was associated with high prevention efficacy of VRC01. This relationship was then mapped to that between PT_80_ at exposure and prevention efficacy, via calculating PT_80_ as the ratio of concentration and geometric mean IC_80_ against all exposing viruses isolated from AMP participants who acquired HIV-1 during the study. Consistent with what was reported in Gilbert et al.,^12^ we found that higher PT_80_ at exposure was associated with higher prevention efficacy of VRC01, with the difference that the former analysis was based on the IC_80_ correlate and used the average concentration over time as a representative concentration at exposure to convert IC_80_ to PT_80_, whereas our analysis was based on the concentration correlate and used the average IC_80_ against all acquired viruses as a representative IC80 to convert concentration to PT_80_. The fact that the same level of PT_80_ corresponded to a lower predicted prevention efficacy in the current analysis, as compared to that in Gilbert et al.,^12^ suggests that variability in PE was likely contributed more by variability in IC_80_ than that in serum concentration of VRC01, and there was considerable variability in concentration (over time) at exposure.

Based on these findings of higher serum concentration of VRC01 being associated with decreased likelihood of HIV-1 acquisition and higher prevention efficacy, we hypothesized that prolonging the elimination phase for slower decay of serum concentrations could enable longer term protection against HIV-1 acquisition. To accomplish this, LS modifications (leucine serine mutation) are being engineered into the next generation of bnAbs for clinical trials to increase the antibody binding affinity for the Fc neonatal receptor (FcRn).^24^ Moreover, since we also found a relationship between body weight and bnAb clearance, future bnAb regimens could consider fixed dose levels based on weight ranges to optimize maintenance of the protective concentrations over the duration of the prevention interval. For future work, a concentration sieve analysis that includes biophysical antibody dynamics (e.g., binding and off-rate) to relevant circulating HIV-1 envelope proteins may improve precision in characterizing the association between HIV-1 acquisition or prevention efficacy and bnAb concentrations measured by binding assays. Additional insights may also be gained with mathematical modelling that is built upon antibody PK and potency combined with HIV-1 viral dynamics; such analyses could help identify whether breakthrough infections are the result of viral genetic resistance to the infused bnAb or whether the limited potency is against all strains of virus.^39^

We also found an inverse association between participants' body weight and HIV-1 acquisition likelihood in the placebo group, where placebo participants with lower body weight had a higher likelihood of HIV-1 acquisition. This association was also true in the VRC01 group, where on average VRC01 recipients who acquired HIV-1 tended to have lower body weight than VRC01 recipients who remained without HIV-1 during the study. Such inverse association remained present after adjusting for demographics and a risk score to account for potential confounding effects. We hypothesized that the observed inverse association between body weight and likelihood of HIV-1 acquisition may partly be caused by other unobserved factors that influence one's general health and immune system and in turn influence likelihood of HIV-1 acquisition (e.g., nutrition),^40^, ^41^, ^42^ or non-sexual behaviours that were not included in the construction of the risk score but could be associated with likelihood of HIV-1 acquisition (e.g., drug use).^43^, ^44^, ^45^ Of note, for a given individual peak VRC01 serum concentrations at any time after each infusion were proportionate to their body weight due to the body weight-based dosing in AMP. Therefore, although the inverse association between body weight and HIV-1 in the placebo group is independent of the presence of VRC01, the same inverse association in the VRC01 group would also lead to the correlates trend that on average VRC01 recipients who acquired HIV-1 tended to have a lower peak concentration than VRC01 recipients without HIV-1. Due to this, body weight was considered a potential confounder in the concentration correlates analysis described above. Although the estimated halved likelihood of HIV-1 acquisition per 10-fold increase in VRC01 concentration^12^ was only slightly dampened after adjusting for body weight in the correlates analysis, our new finding that total body weight was a potential confounder in the correlates analysis has important implications in the design and analysis of future bnAb trials. Specifically, we recommended considering total body weight as a potential predictor of likelihood for HIV-1 acquisition. We also suggest adjusting for total body weight in the correlates analysis of future bnAb trials is warranted.

Lastly, we found in simulation studies that fixed dosing of both VRC01 and a triple bnAb combination provided comparable preventive efficacy as body weight-based dosing on a population-level. Of note, such comparativeness of the two dosing regimens is not necessarily true on an individual level. Specifically, individuals of body weight above the average weight in the study population would receive a higher amount of bnAbs under the weight-based vs. fixed dosing. Consequently, in the context of VRC01 serum concentration being an inverse correlate of HIV-1 acquisition likelihood, individuals that have high body weight are predicted to have a lower likelihood of HIV-1 acquisition under the weight-based vs. fixed dosing. On the other hand, individuals of body weight below the average weight are predicted to have a higher likelihood of HIV-1 acquisition under the weight-based vs. fixed dosing. Therefore, on a population level the two different dosing regimens are expected to have comparable efficacy as the overall efficacy of a regimen is aggregated over individuals of different body weights. Future work on the identification of protective PT_80_ threshold for combination bnAbs is needed to determine whether fixed or body-weight dosing would provide sufficient protection against circulating strains on an individual level for persons above or below the average body weight.

Research on monoclonal antibodies used in oncology found that well-selected fixed dosing was effective while substantially reducing costs and operational complexity.^30^^,^^46^^,^^47^ Fixed dose oncology bnAbs have been approved in Europe.^48^ Various monoclonal antibodies used for SARS-CoV-2 treatment are also administered using fixed dosing, which helped mitigate health care implementation challenges.^49^ For HIV-1 prevention, an ongoing HVTN clinical trial (HVTN 140/HPTN 101, NCT05184452) is evaluating fixed versus body weight-based dosing for comparison of PK parameters and potential use of fixed dosing in future trials. Results from this trial, coupled with additional simulation studies could potentially provide further insights in the comparativeness in prevention efficacy between the weight-based and fixed dosing regimens. Ultimately, an optimal fixed dose may vary by the body weight distribution in different populations, globally. Furthermore, depending on a given monoclonal antibody's therapeutic index and the effect of body weight on its volume of distribution and clearance, multiple fixed doses stratified by body weight cohorts may be most appropriate to harness the advantages of fixed dosing while optimizing safety and efficacy for individuals with comparatively low or high body weight. If fixed dosing is confirmed as an effective strategy for bnAbs that prevent HIV-1, it will decrease the operational burden, thus making use easier, more convenient, and cheaper to administer than body weight-based dosing.

As with previous work assessing the AMP trials,^12^^,^^17^^,^^18^ we assert that bnAb immunoprophylaxis holds promise for HIV-1 prevention, even as improvements in bnAb design are critically needed. To improve upon the AMP outcomes, combinations of bnAbs with multiple specificities may provide the breadth and potency needed to achieve high efficacy to prevent HIV-1 acquisition. Previous research showed that the triple bnAb combination VRC07-523LS, PGT121LS and PGDM1400LS at 40 mg/kg each administered every 16 weeks would have a predicted prevention efficacy of over 95% against both clade C and clade B viruses isolated from AMP placebo recipients who acquired HIV-1 during the trial.^12^ Several clinical trials are now underway to examine safety, tolerability, PK and neutralization of dual antibody combinations (e.g., NCT04173819: 3BNC117-LS-J and 10-1074-LS-J; NCT03928821: VRC07-523LS with PGT121, PGDM1400 or PGT121; NCT04212091: PGT121.414.LS and VRC07-523LS) and triple antibody combinations (e.g., NCT03928821: VRC07-523LS, PGT121 and PGDM1400; NCT05184452: VRC07-523LS, PGT121.414.LS and PGDM1400 LS), along with modifications of antibodies to extend the *in vivo* half-life (reviewed in Miner et al.).^10^ Evidence from prior combination trials indicates that PK of the individual bnAbs is not changed when bnAbs are given in combination,^14^^,^^15^ demonstrating that the described concentration correlates results for a single bnAb can potentially be extended to rank order candidate combination bnAb regimens for future efficacy testing. Ongoing studies are examining the PK of triple combinations of antibodies with non-overlapping viral epitope specificities that complement each other to maximize both antiviral potency at lower concentrations and breadth of coverage for circulating strains. Antibodies engineered to improve tissue penetration, enhance antibody Fc-effector functions, extend half-life, increase potency, and improve formulation (facilitating alternative administration routes, such as subcutaneous or intramuscular injection) are needed for optimal effectiveness.

To improve bnAb regimens in the future, additional work is needed to measure the concentrations of infused bnAbs in mucosal tissue and how they correlate with serum concentrations is important for understanding the mechanisms of protective immunity and improving upon bnAb immunoprophylaxis strategies.^50^ Astronomo et al.^50^ reported that IV administered VRC01 distributes to the female genital and male rectal mucosa. These VRC01 concentrations present in the mucosal tissue were high enough to maintain neutralising activity, supporting a role for the presence of bnAb in the mucosal tissues in preventing HIV-1 acquisition. IV-administered antibodies demonstrated two phases of distribution. Within a day, the submucosa is penetrated, with the stratum corneum requiring approximately a week to reach saturation^36^ revealing that Ab levels circulating in the blood can be at a different concentration from that of mucosal tissue depending on the timing post infusion. Given the relatively high concentration of the Fc neonatal receptor (FcRn) in mucosa, the serum-mucosa concentration correlations for LS-modified bnAbs (with increased FcRn affinity) are likely to differ from those in non-LS-modified bnAbs. Thus, additional studies are needed to determine the correlation of Ab levels in the blood vs. mucosa (particularly for LS-modified antibodies) and the threshold for prevention at mucosal routes of entry.

There are two main limitations of our study. First, due to the relatively sparse 4-weekly sampling of the VRC01 serum concentration time-points, there is limited precision in the estimated concentration especially during the distribution phase of VRC01 shortly after each infusion. This added variability in the assessment of concentration as a correlate of HIV-1 acquisition. The second main study limitation is that one of the core assumptions of our timing methodology is that the viral population is sampled prior to the onset of host adaptive immunity.^37^^,^^51^ The Poisson model used for sequence-based estimation of acquisition time is generally robust to small deviations from this assumption, i.e., the very beginning of selection pressure at a single T-cell epitope for example. However, using sequence data generated from early HIV-1 acquisition in the RV217^52^ and FRESH^53^ studies, we have seen that in some instances, for sequences sampled later after acquisition, typically two to three months after the last study visit without HIV-1, our sequence-based time estimator tends to under-estimate the true time of acquisition (Rossenkhan et al., unpublished), as bnAb concentration is highest early within infusion cycles. Consequently, having biased-high estimates of concentrations at exposure times when compared to the average concentration over time would lead to a weaker concentration correlate, assuming exposures to HIV-1 occur approximately uniformly over time, and applying different timing estimate methods could subsequently affect the correlation estimates.

In conclusion, we found that serum VRC01 concentration was inversely correlated with HIV-1 acquisition likelihood after accounting for VRC01 recipients’ body weight and were positively correlated with prevention efficacy, suggesting that bnAb serum concentration may be a useful marker for dosing regimen selection. In addition, future bnAb trials could consider fixed dosing, as opposed to body weight-based dosing to reduce cost and operational complexity of administration.

## Contributors

KES, YH, SK, LC, MSC, PBG, GDT conceived and designed the study; KES, YH, PBG, GDT supervised the data analyses, interpreted the data, and verified the underlying data; KES, JRH, NLY, GDT developed method and oversaw pharmacokinetic data generation; JRH, CB, KC, MR developed methods and performed experiments; MS, Lu Z, SS managed and analysed laboratory data; LZ, ER, YH, AK performed data analysis, and data interpretation; ACd, MJ, PBG oversaw and verified the underlying clinical data; AR, JH, MJM oversaw study management through clinical, laboratory and analytical stages; MSK oversaw laboratory quality assurance; ABM developed method and assay concordance; CW and JIM provided data and reagents; JRM provided critical reagents; PTE, EEG, and RR developed and ran the infection timing analyses; MEA and JAW conducted the anti-drug antibodies analysis; PA, JAH, JC, FL, CO, IF, PG, SE, NM, LC, LM, DM, MSC designed the clinical trials and/or enrolled participants at clinical sites; KES, YH, PBG, GDT drafted the manuscript; HA contributed, edited, and reviewed this manuscript; and all authors contributed, reviewed and approved the final version of the manuscript. The corresponding authors had full access to all the data in the study and had final responsibility for the decision to submit for publication.

## Data sharing statement

All data will be made available upon request.

## Declaration of interests

Kelly E Seaton - support for meetings, HIV Vaccine Trials Network (HVTN).

M Julianna McElrath - support for Keystone Symposia.

Ian Frank - consulting fees from Gilead, ViiV Health Care and grants or contracts from Moderna, Janssen Therapeutics, Pfizer.
