# Supplementary material for: Pharmacokinetic serum concentrations of VRC01 correlate with prevention of HIV-1 acquisition
Source: eBioMedicine. 2023 Jun 8;93:104590. doi: 10.1016/j.ebiom.2023.104590 (PMC10363420; doi:10.1016/j.ebiom.2023.104590)

Supplemental Materials

PK BAMA Assay

During the conduct of the PK BAMA assay, NeutrAvidin (ThermoFisher, Waltham, MA) was covalently coupled to the surface of activated-carboxylated magnetic microspheres (Luminex Corp, Austin, TX) through an intermediate Sulfo-NHS (N-hydroxysulfosuccinimide) ester bond. NeutrAvidin microspheres were then bound to a biotinylated goat anti-mouse antibody cross adsorbed against human immunoglobulins (5 micrograms per 1 million microspheres), (SouthernBiotech, Birmingham, AL). The beads were then incubated with anti-VRC01 idiotype monoclonal antibody (5C9 IgG2A anti-ID) (NIH, Bethesda, MD) for 30-60 minutes, washed, and resuspended in BAMA wash buffer until used in the assay.

Controls and human sera titrations were prepared using assay diluent [1% Milk Blotto (w/v), 5% Normal Goat Sera (v/v) and 0·05% Tween-20 (v/v) in Phosphate Buffered Saline (PBS)] in duplicate wells and incubated with prepared capture microspheres at 22°C for two hours. Assay plates were washed three times with BAMA Wash Buffer (1% Bovine Serum Albumin (w/v), 0·05% Tween-20 (v/v) and 0·05% Sodium Azide (w/v) in PBS) and added with a goat anti- human IgG detection antibody conjugated to R-phycoerythrin. Assay plates were analyzed with BioPlex Manager, Version 6·1, security edition on Bio-Plex 200 Systems (Bio-Rad, Hercules, CA). All assays were performed in compliance with Good Clinical Laboratory Practice (GCLP) standards, including Levey-Jennings tracking charts for control curves and electronic capture and storage of raw data.

Commercially available HIV-1 seronegative samples (Bioreclamation IVT) were used to establish the lower limit of detection (LOD) and lower limit of quantitation (LLOQ) of VRC01 in human serum. Thirty-three known HIV-1 seronegative samples were tested for VRC01 in a binding antibody multiplex assay. We used the 95th percentile of the fluorescence intensity background subtracted (MFI) and the mean MFI plus 3.3 standard deviations to establish the LOD. From the qualification experiment, entering the mean MFI + 3.3 standard deviations (1729) of the known seronegative samples into the inverted 5PL equation established the concentration LOD as 339 pg/ml, while we used the 95th percentile MFI of 1660 as the MFI LOD. We established the LLOQ at 686 pg/ml, determined as the least concentrated point on the standard curve above the LOD that recovered accurately. Accurate recovery is determined as the observed concentration being within 30% of the expected concentration. We established the upper limit of quantitation (ULOQ) as the minimum of 18,000 MFI and the MFI value of the highest expected concentration of the standard curve that accurately recovered.

Samples with an MFI or in-well concentration less than the respective LOD were assigned < LOD, samples with an in-well concentration less than the LLOQ were assigned < LLOQ, and samples above the ULOQ were assigned > ULOQ. The selection and positivity method proceeds as follows: (1) compare sample in-well concentrations and MFIs to the previously established LODs to assign one of four curve locations, (2) group samples by curve location, preferring samples LR, then < LLOQ, then < LOD, (3) of the samples assigned LR, select the sample with the in-well concentration closest to the inflection point of the respective standard curve, (4) if no samples assigned LR, select a sample < LLOQ at the least diluted, (5) if no samples < LLOQ, select a sample < LOD at the least diluted, (6) samples selected to report with curve location < LLOQ or LR, assign positive, and (7) samples selected to report with curve location < LOD, assign negative.

We established the upper limit of quantitation (ULOQ) as the minimum of 18,000 Mean Fluorescence Intensity units (MFI) or the MFI value of the highest concentration of the standard curve that was accurately recovered (70-130% of expected value). Assay controls included a standard curve and three positive controls spiked with 0·008, 0·002, and 0·0005 µg/ml of VRC01 mAb on each assay plate. For samples on a given plate to be acceptable for reporting, multiple conditions had to be met: (1) 2/3 consecutive spiked controls must recover accurately, (2) the area under the curve and half maximal effective concentration (EC50) of the standard curve must fall within three standard deviations of historical data in Levey-Jennings tracking charts, (3) if the high spiked control failed to recover, the in-well sample concentrations must be below 0·002 µg/ml, (4) if the low spiked control failed to recover, the in-well sample concentration must be above 0·0005 µg/ml, (5) the CV of samples with concentrations above the LOD must be less than 20%, and (6) the sample MFI must be below the ULOQ.

Supplemental Figures

Supplemental Figure 1. PK-BAMA Assay Qualification/Validation Results.

Supplemental Figure 2a-d. The distribution of the individual-level (a) clearance from the central compartment (CL, L/day), (b) Volume of the peripheral compartment (Vp, L), (c) Elimination half-life (days), (d) Distribution half-life (days) over the 10 infusion intervals, based on base population PK model (n=189).

Supplemental Figure 3a-b: Anti-drug antibodies Tier II confirmation among VRC01 Recipients who remained without HIV during the study in (a) HVTN 704/HPTN 085 (n=100) and (b) HVTN 703/HPTN 081 (n=100).

Supplemental Figure 4. Population PK estimates of VRC01 from the final model. %RSE: relative standard error of the fixed effects estimate, calculated as (standard error of the estimate divided by the point estimate)*100. %CV: coefficient of variation for random effect estimates.

Supplemental Figure 5a-c. Goodness-of-fit model diagnostic plots obtained from the final population PK model. (a) Observations vs. individual predictions on log-scale, (b) Scatter visual prediction check (VPC) that shows how well the observed data (points) align with the final popPK model simulated, (c) Percentile VPC that shows how well the observed data align with the final popPK model simulated percentiles at each binned time-point (n=189).

Supplemental Figure 6a-b. (a) Distribution half-life, (b) Elimination half-life at pre-acquisition time-points does not differ between VRC01 recipients who acquired HIV-1 (n=107) vs. VRC01 recipients without HIV-1 (n=82) by dosing regimen. Estimated distribution/elimination half-life is shown in red for VRC01 recipients who acquired HIV-1 and in blue for VRC01 recipients without HIV-1 for low (10 mg/kg) and high (30 mg/kg) dose groups combined across the two AMP trials. Box and whiskers plots show the median, 25th and 75th percentiles.

Supplemental Figure 7. Participants' total body weight association with likelihood of HIV-1 acquisition by study. Hazard ratio of HIV-1 acquisition per 5 kg increase in participants' baseline body weight was estimated via Cox regression models by study, treatment group and dose (n=4611).

Supplemental Figure 8a-b. Controlled risk of HIV-1 acquisition by day 595 as a function of body weight (kg) for (a) HVTN703/HPTN081 (n=1924) and (b) HVTN704/HPTN085 (n=2687).

Supplemental Figure 9a-b: Predicted prevention efficacy of fixed dose versus body weight-dose of the triple bnAb regimen PGT121LS + PGDM1400LS + VRC07-523LS and of VRC01 at steady state against (a) HVTN703/HPTN 081 placebo viruses (n = 47) and (b) HVTN704/HPTN 085 placebo viruses (n = 70) circulating in each AMP trial. All predictions were made under the scenario that PGT121LS and PGDM1400LS have 2.5-times higher half-lives than PGT121 and PGDM1400, based on modelling of the observed serum concentration data of PGT121[27] and PGDM1400 [19]. Predicted prevention efficacy at steady state was based on prevention efficacy vs. PT80 curve in AMP[12] and Pegu et al.[5]. Solid line: median. Shaded area: 95% prediction interval.

Supplemental Figure 1: PK-BAMA Assay Qualification/Validation Results.

Parameter	Qualification Results	Validation Results
**Accuracy: Standard Curve**	Nine titration points in the standard curve had an observed concentration between 70-130% of the expected value	Eleven titration points in the standard curves had an observed concentration between 70-130% of the expected value
**Accuracy: Spiked Samples**	100% (3/3) Spiked QC samples (0.0005 µg/ml, 0.0020 µg/ml, and 0.0080 µg/ml) had an observed concentration between 70-130% of the expected value.	100% (3/3) Spiked QC samples (0.0005 µg/ml, 0.0020 mcg/ml, and 0.0080 mcg/ml) had an observed concentration between 70-130% of the expected value.
**Specificity: Non-Specific Background**	Non-specific background was estimated at 6.67% based on the population tested in qualification (N=30)	Sample MFI values (N=32) were qualitatively 97% concordant with VRC01 IgG negative status
**Specificity: Positivity**	97% of spiked samples were concordant with VRC01 IgG positive status	100% of spiked samples were concordant with VRC01 IgG positive status
**Precision: Repeatability**	MFI output of each dilution point above the LOD from 10 standard curve replicates had CV<27%	MFI output of each dilution point above the LOD from 10 standard curve replicates had % CV less than 3.8%
**Precision: Intermediate Precision (Operator to Operator and Day to Day)**	Standard curve MFIs had %CV less than 27% (day-to-day) and 28% (operator-to-operator) across all dilution points above the LOD.	Standard curve MFIs had %CV less than 6.1% (day-to-day) and 4.6% (operator-to-operator) across all dilution points above the LOD.
**Limits of Detection (LOD) and Quantitation (LLOQ)**	The determined assay LOD is 0.000387 µg/ml and the assay LLOQ is 0.000686 µg /ml in 1:100 diluted seronegative serum. This translates to a physiologic LOD of 0.0387 µg/ml and physiologic LLOQ of 0.0686 µg/ml.	The determined assay LOD is 0.00058 µg/ml and the assay LLOQ is 0.000686 µg /ml in 1:100 diluted seronegative serum. This translates to a physiologic LOD of 0.058 µg/ml and physiologic LLOQ of 0.0686 µg/ml.

Supplemental Figure 2a-d. The distribution of the individual level (a) clearance from the central compartment (CL, L/day), (b) Volume of the peripheral compartment (Vp, L), (c) Elimination half-life (days), and (d) Distribution half-life (days) over the 10 infusion intervals, based on base model.

(a) Distribution of the individual level CL

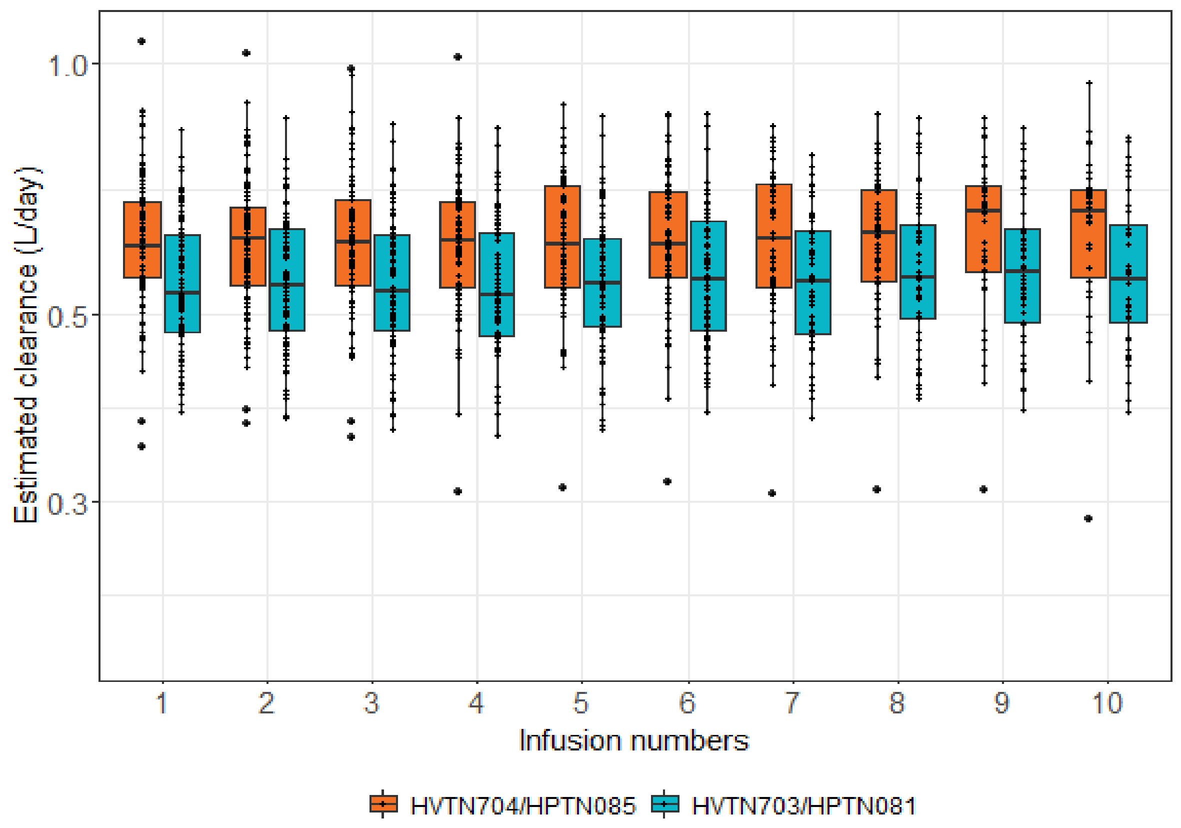

(b) Distribution of the individual level Vp

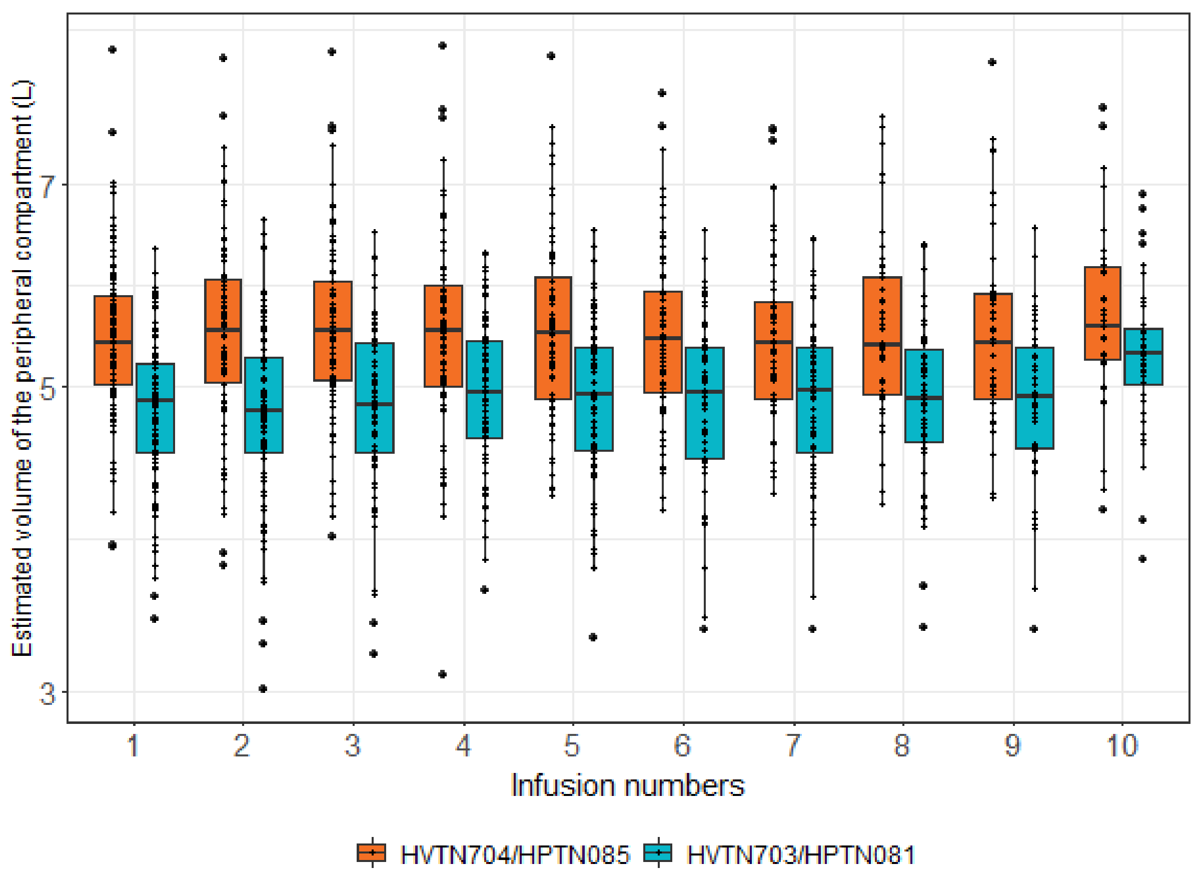

(c) Distribution of the individual level elimination half-life

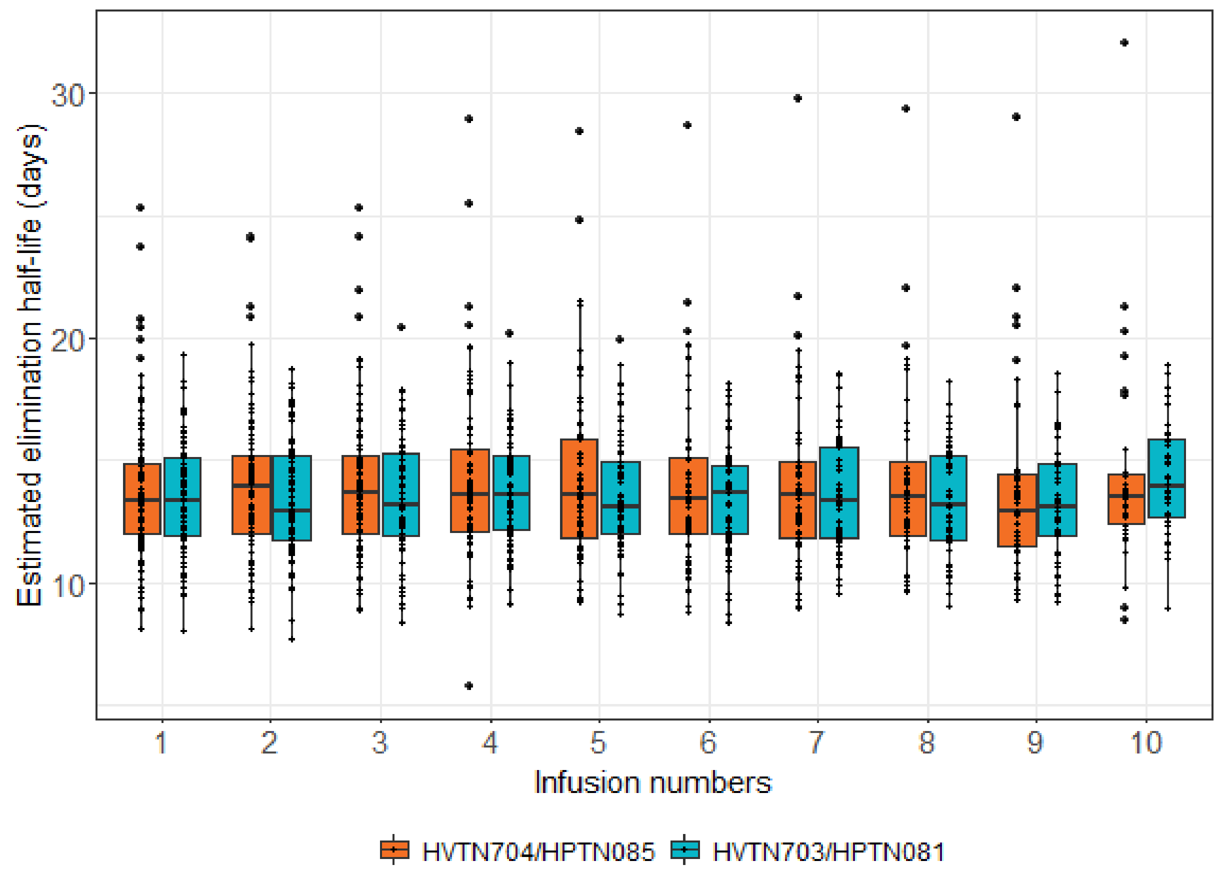

(d) Distribution of the individual level distribution half-life

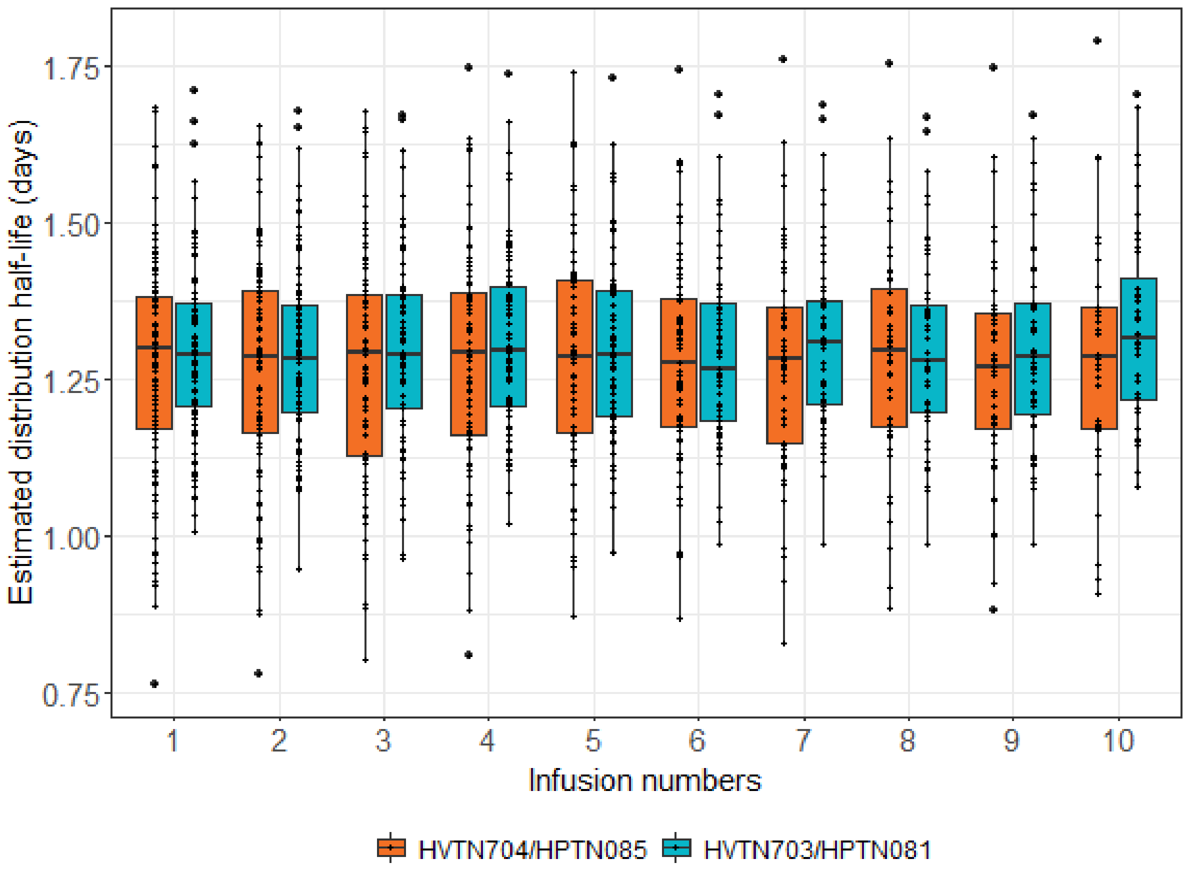

Supplemental Figure 3a-b: Anti-drug antibodies Tier II confirmation among VRC01 Recipients who remained without HIV during the study in (a) HVTN 704/HPTN 085 (n=100) and (b) HVTN 703/HPTN 081 (n=100).

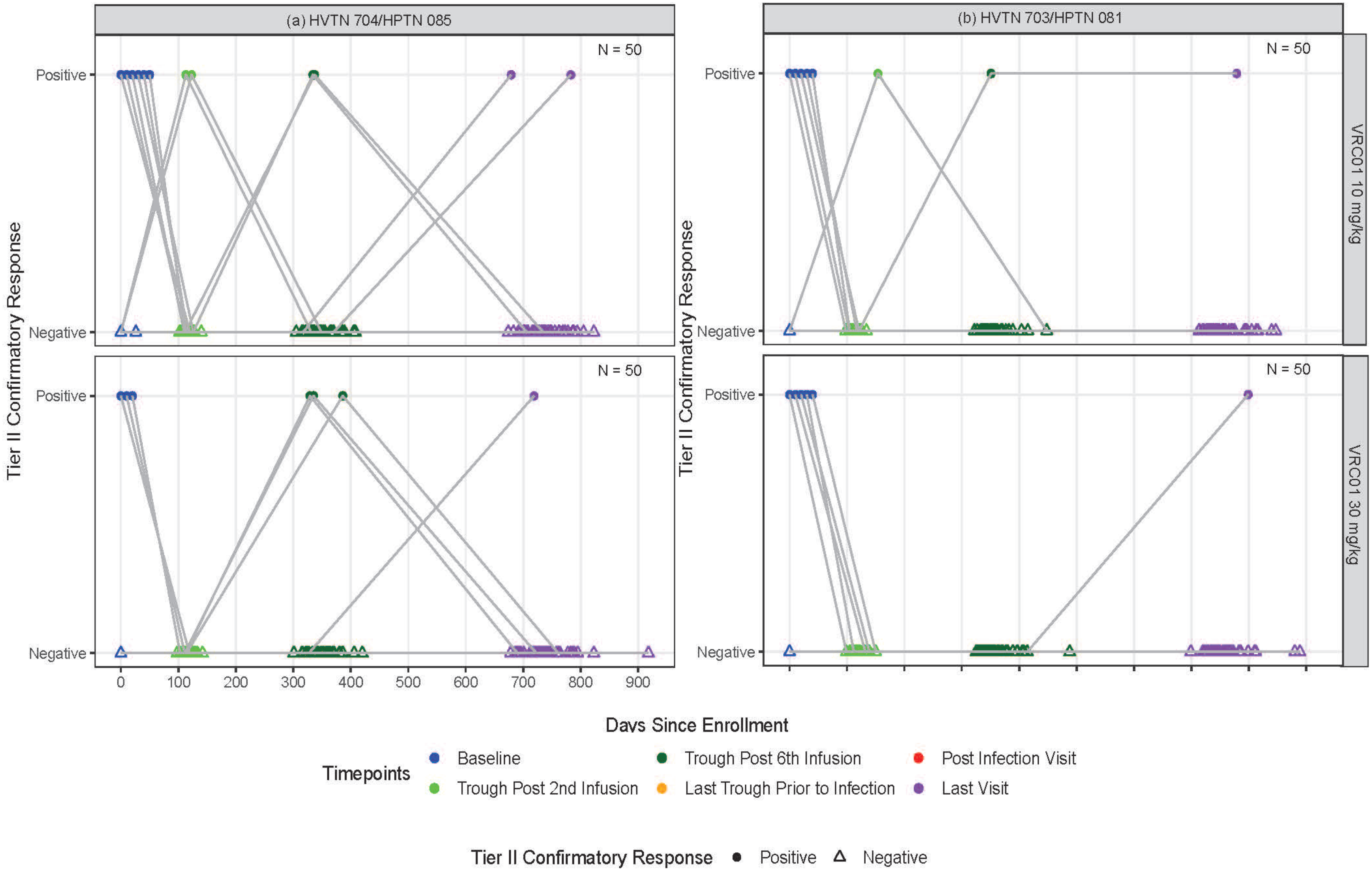

Supplemental Figure 4. Population PK estimates of VRC01 from the final model. %RSE: relative standard error of the fixed effects estimate, calculated as (standard error of the estimate divided by the point estimate)*100. %CV: coefficient of variation for random effect estimates.

	Parameter	Estimate	%RSE	95% CI LBOUND	95% CI UBOUND	%CV
**Fixed effects**	CL (L/day): clearance from the central compartment	0.575	2.06	0.552	0.598	–
	Body weight influence on CL	0.331	21.39	0.193	0.470	–
	Study influence on CL	0.111	24.99	0.057	0.165	–
	Vc (L): volume of the central compartment	3.532	4.46	3.223	3.840	–
	Q (L/day): inter-compartmental distribution clearance	0.676	1.93	0.650	0.701	–
	Vp (L): volume of the peripheral compartment	4.749	2.12	4.552	4.946	–
	Study influence on Vp	0.165	16.41	0.112	0.218	–
**Standard Deviation of the Random Effects**	CL (L/day): clearance from the central compartment	0.172	5.62	0.153	0.191	17.19%
	Vc (L): volume of the central compartment	0.248	14.80	0.176	0.320	24.77%
	Q (L/day): inter-compartmental distribution clearance	0.064	19.56	0.039	0.088	6.38%
	Vp (L): volume of the peripheral compartment	0.145	7.97	0.123	0.168	14.55%
**Error Model Parameters**	Residual error (proportional)	0.188	1.48	0.183	0.194	18.81%

Supplemental Figure 5. Goodness-of-fit model diagnostic plots obtained from the final population PK model. (a) Observations vs. individual predictions on log-scale, (b) Scatter visual prediction check (VPC) that shows how well the observed data (points) align with the final popPK model simulated, (c) Percentile VPC that shows how well the observed data align with the final popPK model simulated percentiles at each binned time-point (n=189).

a) Observations vs. Individual Predictions on log-scale

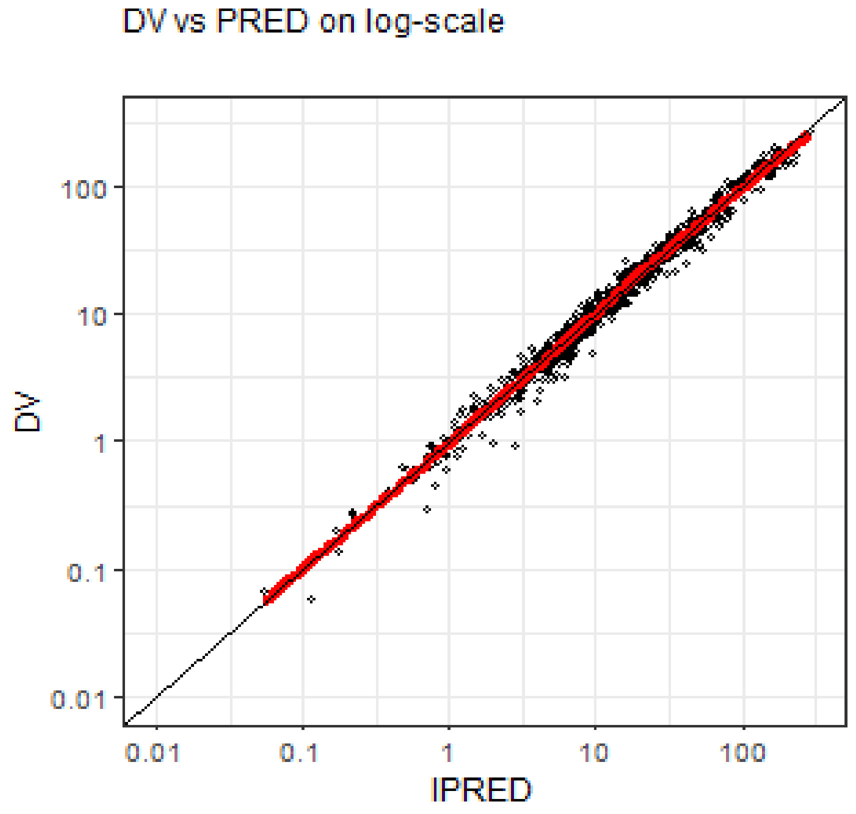

b) Scatter Visual Predictive Checks (VPC)

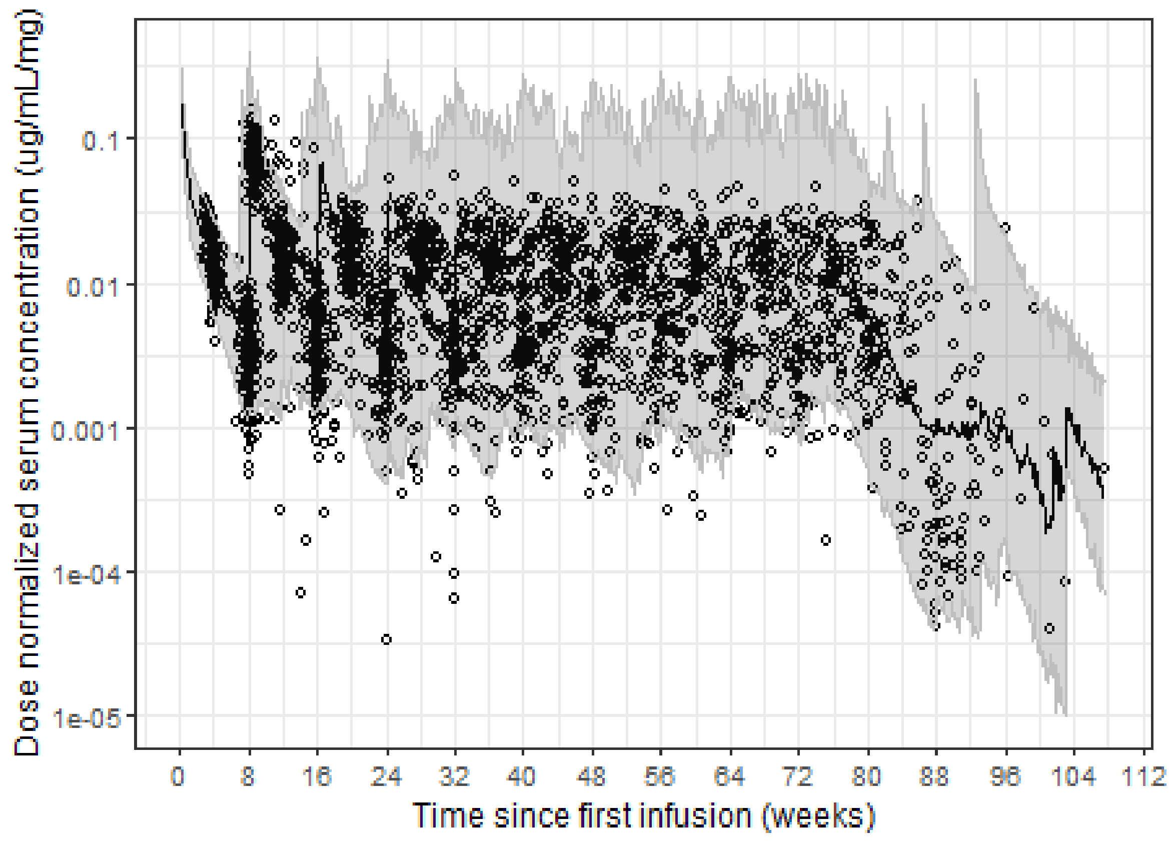

c) Percentile Visual Predictive Checks (VPC)

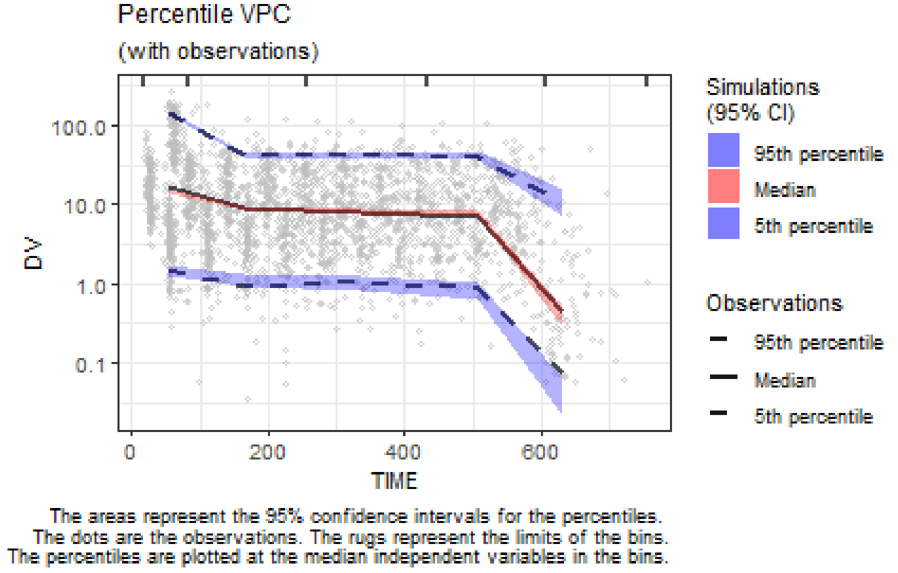

Supplemental Figure 6a-b. (a) Distribution half-life, (b) Elimination half-life at pre-acquisition time-points does not differ between VRC01 recipients who acquired HIV-1 (n=107) vs. VRC01 recipients without HIV-1 (n=82) by dosing regimen. Estimated distribution/elimination half-life is shown in red for VRC01 recipients who acquired HIV-1 and in blue for VRC01 recipients without HIV-1 for low (10 mg/kg) and high (30 mg/kg) dose groups combined across the two AMP trials. Box and whiskers plots show the median, 25th and 75th percentiles.

(a) Pooled AMP Trials: Distribution

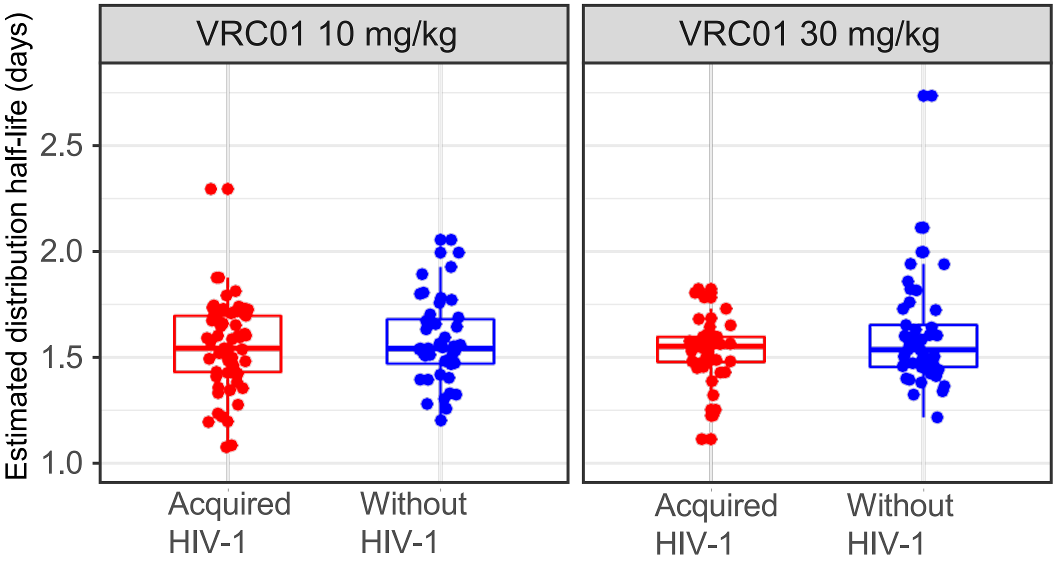

(b) Pooled AMP Trials: Elimination

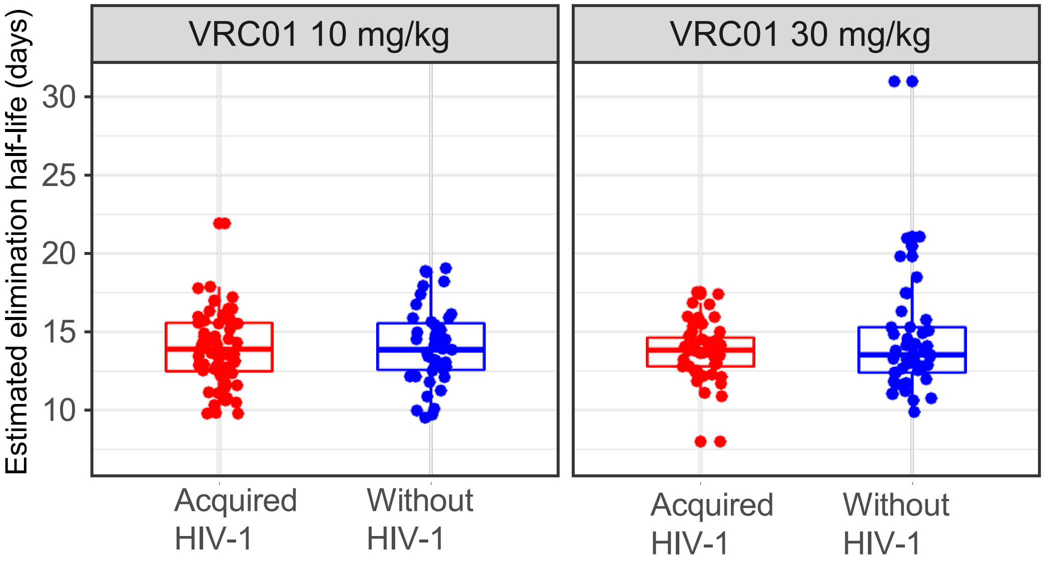

Supplemental Figure 7. Participants' total body weight association with likelihood of HIV-1 acquisition by study. Hazard ratio of HIV-1 acquisition per 5 kg increase in participants' baseline body weight was estimated via Cox regression models by study, treatment group and dose (n=4611).

Trial and Treatment	No. diagnosed with HIV-1	HR of HIV−1 acquisition per 5 kg increase in body weight (95% CI)	P Value
HVTN704/HPTN085			
Control	38	0.76 (0.67 to 0.87)	<0.001
VRC01 Pooled	60	0.82 (0.74 to 0.91)	<0.001
VRC01 10 mg/kg	32	0.84 (0.73 to 0.96)	0.009
VRC01 30 mg/kg	28	0.80 (0.69 to 0.94)	0.006
HVTN703/HPTN081			
Control	29	0.91 (0.80 to 1.05)	0.204
VRC01 Pooled	47	0.90 (0.80 to 1.01)	0.068
VRC01 10 mg/kg	28	0.94 (0.81 to 1.08)	0.383
VRC01 30 mg/kg	19	0.84 (0.70 to 1.02)	0.074

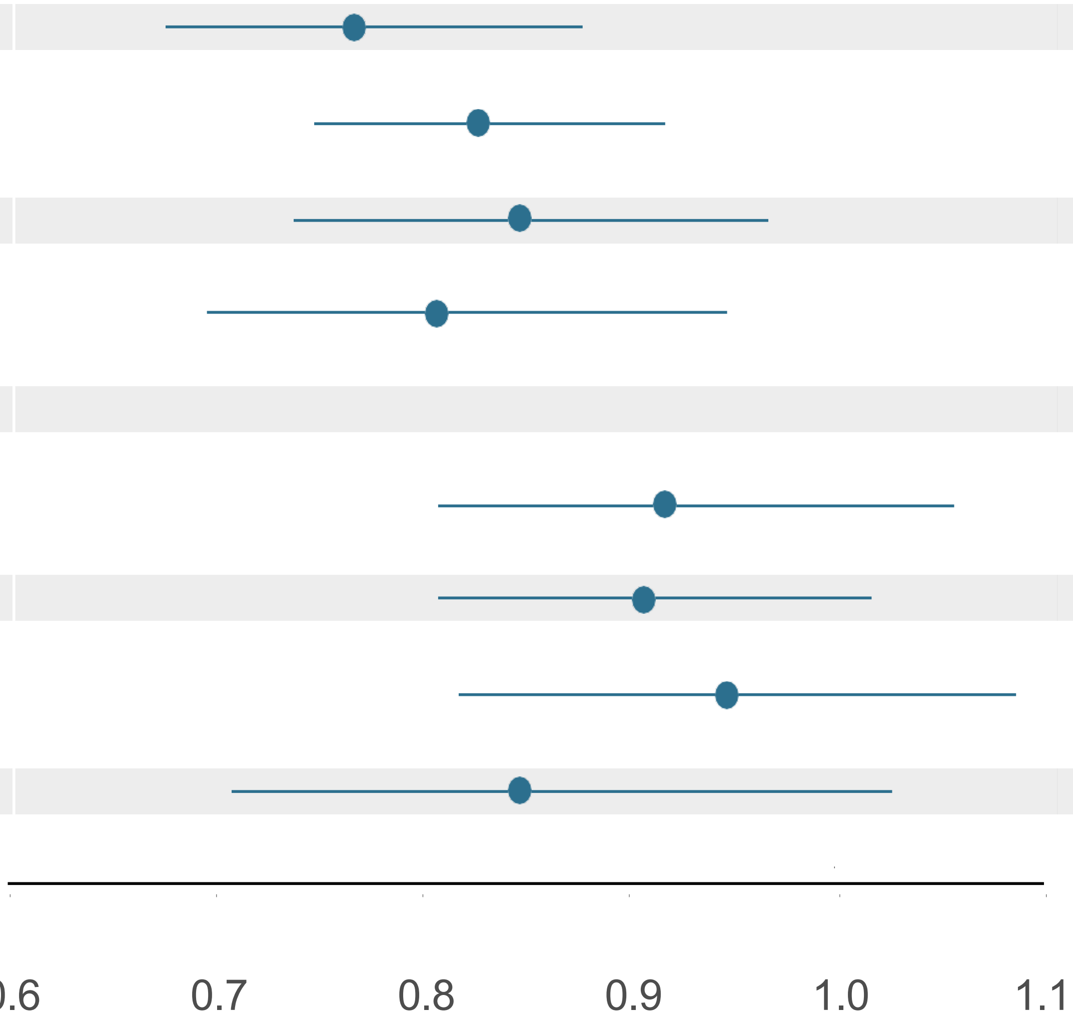

Supplemental Figure 8: Controlled likelihood of HIV-1 acquisition by day 595 as a function of body weight (kg) for (a) HVTN703/HPTN081 (n=1924) and (b) HVTN704/HPTN085 (n=2687).

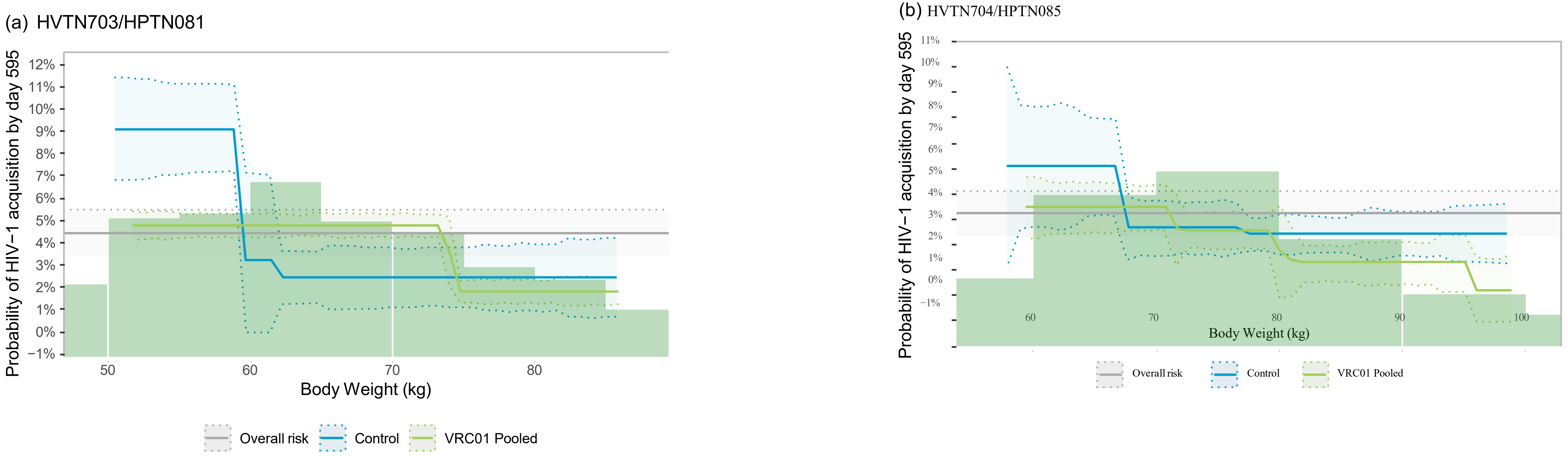

Supplemental Figure 9a-b: Predicted prevention efficacy of fixed dose versus body weight-dose of the triple bnAb regimen PGT121LS + PGDM1400LS + VRC07-523LS and of VRC01 at steady state against (a) HVTN703/HPTN 081 placebo viruses (n = 47) and (b) HVTN704/HPTN 085 placebo viruses (n = 70) circulating in each AMP trial. All predictions were made under the scenario that PGT121LS and PGDM1400LS have 2.5-times higher half-lives than PGT121 and PGDM1400, based on modelling of the observed serum concentration data of PGT121[27] and PGDM1400 [19]. Predicted prevention efficacy at steady state was based on prevention efficacy vs. PT80 curve in AMP[12] and Pegu et al.[5]. Solid line: median. Shaded area: 95% prediction interval.

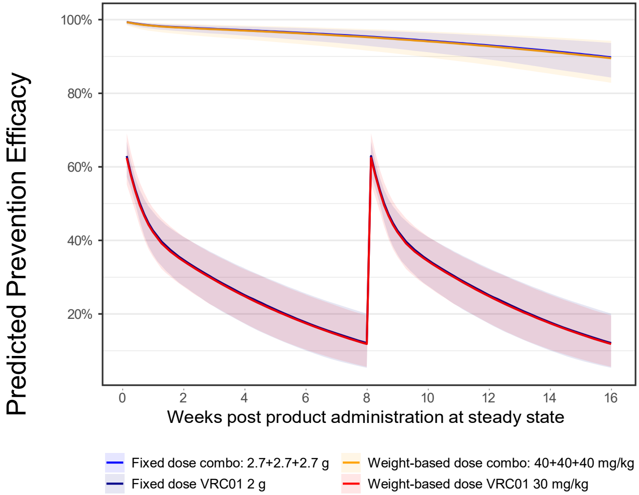

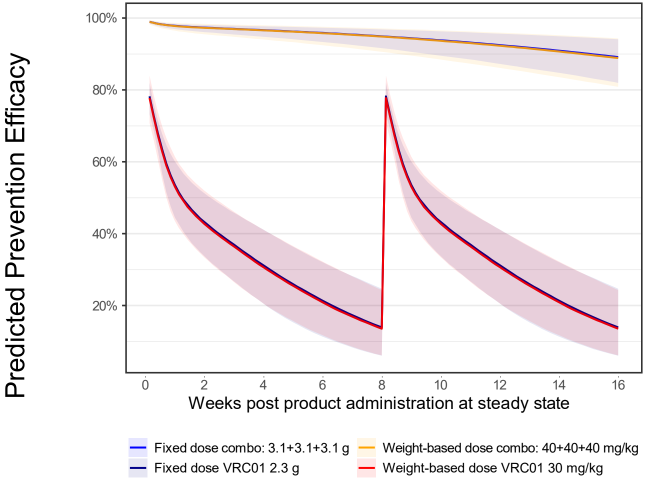

Supplement: Supplemental Material and Figures S1–S9 [file mmc1.pdf]
